# Essentiality-driven prediction of anticancer drug responses in preclinical and clinical contexts

**DOI:** 10.1016/j.isci.2026.116980

**Published:** 2026-07-27

**Authors:** Hongtu Cui, Xiaohui Du, Haixia Guo, Zhen Liao, Bingying Wang, Xiang Lian, Ji-Yun Zhang, Xing Lu, Dongdong Zhang, Anqiang Ye, Jing Feng, Jing Li, Zhenshun Cheng, Feng-Biao Guo

**Affiliations:** 1 Department of Respiratory and Critical Care Medicine, Zhongnan Hospital of Wuhan University, School of Pharmaceutical Sciences, Wuhan University, Wuhan, China; 2 Key Laboratory of Combinatorial Biosynthesis and Drug Discovery, Ministry of Education, Wuhan University, Wuhan, China; 3 School of Cyber Science and Engineering, Wuhan University, Wuhan, China; 4 School of Artificial Intelligence, Wuhan University, Wuhan, China; 5 Department of Precision Medicine, Changhai Hospital, Second Military Medical University (Naval Medical University), Shanghai, China; 6 National Key Laboratory of Immunity and Inflammation, Institute of Immunology, Naval Medical University, Shanghai, China; 7 Department of Respiratory and Critical Care Medicine, Zhongnan Hospital of Wuhan University, Wuhan, China; 8 Wuhan Research Center for Infectious Diseases and Cancer, Chinese Academy of Medical Sciences, Wuhan, China

**Keywords:** gene essentiality, drug response, deep learning, lung cancer, patient survival

## Abstract

Precision oncology relies on tumor molecular profiles to predict drug responses. Instead of using conventional molecular features directly, we construct predictive signatures based on gene essentiality. Here, we present DrGee, an essentiality-centered platform that infers drug sensitivity solely from gene expression profiles. The built-in DeepEEAA model integrates gene expression, gene essentiality, drug-protein affinity, and drug-gene associations to quantitatively predict IC_50_ values. DeepEEAA achieved competitive predictive performance on independent cell line datasets (R^2^ = 0.764; MSE = 0.9345), outperforming recent benchmark deep learning methods. DrGee prioritized four candidate drugs for the 95-D lung cancer cell line, among which BI-97C1 and trimetrexate were validated by *in vitro* assays and mouse xenograft experiments. Robust predictive performance was further confirmed in OVCAR8 ovarian cancer cells. In TCGA cohorts, essentiality-driven predictions stratified patients with significantly different overall survival outcomes (AUC-PR = 0.825), highlighting the translational potential of DrGee.

## Highlights

• DeepEEAA predicts IC_50_ for known drugs in new cellular contexts using gene essentiality
• DrGee prioritizes drugs using only gene expression profiles
• *In vitro* and *in vivo* validation support DrGee’s drug prediction accuracy
• Gene essentiality features predict TCGA drug responses and stratify patient survival

## Graphical abstract

**Figure:**
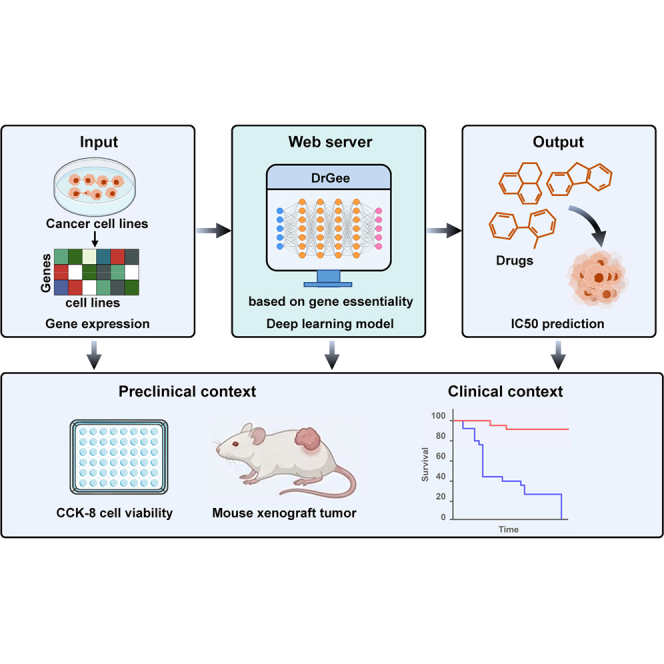

## Teaser

Cancer; Clinical genetics

## Introduction

Cancer remains a leading cause of morbidity and mortality worldwide.^1^^,^^2^ In this context, anticancer drugs remain the cornerstone of clinical treatment. However, two major challenges persist in the clinical application of anticancer drug therapy. First, cancer is characterized by pronounced heterogeneity, and interindividual genomic variation is one of the key factors contributing to substantial differences in patients’ sensitivity to agents and therapeutic outcomes.^3^^,^^4^^,^^5^ Second, drug toxicity remains a major factor compromising patients’ quality of life.^6^ Notably, drug repurposing has emerged as a promising strategy to expedite therapeutic development by leveraging compounds with known safety profiles.^7^^,^^8^ However, accurately predicting tumor-specific drug sensitivity, whether for novel or repurposed agents, constitutes a critical unmet need in precision oncology,^9^ particularly for tumors with limited molecular characterization.^10^

In recent years, large-scale pharmacogenomic resources, including the cancer cell line encyclopedia (CCLE),^4^ cancer target discovery and development (CTD^2^),^11^ genomics of drug sensitivity in cancer database (GDSC),^12^ and the cancer dependency map (DepMap),^13^^,^^14^ have enabled systematic profiling of drug responses across diverse cancer models and stimulated the development of machine-learning-based prediction approaches.^15^^,^^16^^,^^17^^,^^18^ Despite this progress, a substantial proportion of existing methods rely on high-dimensional multi-omics data for model training, which restricts their applicability to clinical samples that are often incompletely characterized and increases translational complexity and cost.^10^ Moreover, many current approaches underutilize gene essentiality, a fundamental determinant of cancer cell survival that is closely linked to drug mechanisms of action and on-target toxicity. Although computational methods for predicting gene essentiality have been developed,^19^^,^^20^ they are frequently not designed for direct integration into drug-response prediction frameworks or depend on multi-modal genomic inputs that limit their suitability for routine clinical use. In contrast, existing drug-response prediction methods^21^^,^^22^ that incorporate gene essentiality typically rely on experimentally measured essentiality profiles as input features, which are costly to obtain and not routinely available in clinical settings, whereas our approach requires only transcriptomic data at inference time by inferring essentiality from expression.

To provide novel and potential solutions for these limitations, we present DrGee (an online server for cancer drug ranking based on gene essentiality derived from expression), an end-to-end platform that prioritizes candidate anticancer drugs using only gene expression data as input. Specifically, DrGee first infers gene essentiality from expression profiles using a computational model, then leverages these inferred essentiality values within DeepEEAA. Central to its design, DrGee relies on DeepEEAA, a deep learning framework that integrates gene expression, gene essentiality, drug-protein affinity, and predicted drug-gene associations to predict drug sensitivity from quantitative biological features. By focusing on essentiality-centered features, the platform is designed to remain applicable to settings in which extensive molecular profiling is unavailable. Through rigorous validation using independent pharmacogenomic datasets, complementary *in vitro* and *in vivo* models, and clinical response data, we show that essentiality-driven modeling supports robust drug-sensitivity prediction with potential clinical relevance across diverse cancer contexts. Collectively, this work presents a scalable grounded artificial intelligence framework that supports personalized anticancer drug prioritization and may inform translational precision oncology strategies.

## Results

### Identification of drug-gene associations using DepMap and assessment of predicted inhibitory associations via the L1000 transcriptomic dataset

**Figure 1:**
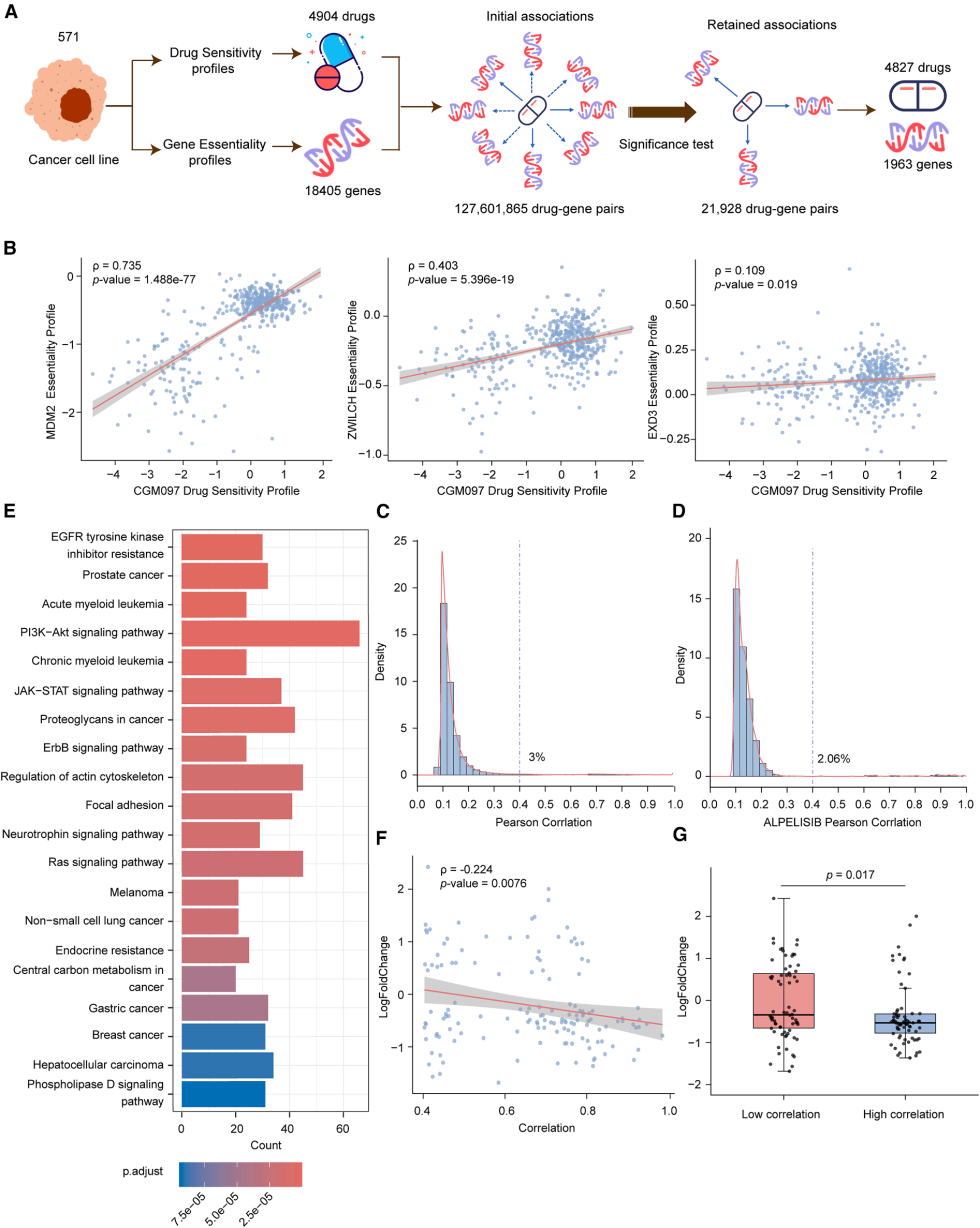
Inferred drug-gene associations from the DepMap database (A) Schematic representation of drug-gene pair identification workflow. (B) Scatterplot of CGM097 drug sensitivity profiles versus *MDM2*, *ZWILCH*, and *EXD3* gene essentiality profiles across 449 cell lines. Correlation coefficients (ρ) and statistical significance (*p* values) of Pearson’s correlation analysis were indicated. Distribution of drug-gene associations among (C) all drugs and (D) individual drug alpelisib. (E) The bar chart depicts the top 20 KEGG-enriched pathways of 1963 genes. (F) Scatterplot illustrating the relationship between correlation and Log2FoldChange across 137 drug-gene pairs, with Pearson’s correlation coefficient (ρ = 0.204) and statistical significance (*p* = 0.078) indicated. (G) Boxplot comparing Log2FoldChange distributions between low and high drug-gene correlation groups (across 137 drug-gene pairs), stratified by median correlation. Boxes indicate the interquartile range (IQR), center lines denote medians, and whiskers extend to 1.5× IQR. Data are represented as mean ± SD. Statistical significance was determined using the Wilcoxon rank-sum test (*p* = 0.017).

We attempted to identify genes associated with drugs in the DepMap,^14^ based on the drugs’ cellular sensitivity and the genes’ cellular essentiality profile across all reference cell lines (Figure 1A). The former is “drug effect,” which assesses a drug’s ability to inhibit cell growth, thereby directly indicating its therapeutic efficacy at the cell level. The latter denotes “gene effect,” which quantifies the impact of knocking out specific genes on cell growth and survival, thus directly reflecting gene essentiality. The sensitivity profile for each drug involves 45 to 750 cell lines, whereas the essentiality profile of each gene accounts for 12 to 754 cell lines. For a given drug-gene pair, we calculated pairwise correlations between these two effects. A significant positive correlation suggests that a drug may be associated with the corresponding gene, as a gene with high essentiality in sensitive cell lines indicates potential dependence on that gene or its pathway for cell survival.

To contextualize this correlation-based approach, we characterized the association patterns between drug sensitivity and gene essentiality using CGM097 and three candidate genes. Notably, *MDM2* is the well-validated authentic target of CGM097. Across cell lines, the correlation coefficient between CGM097’s sensitivity profile and *MDM2*’s essentiality profile was 0.735 (*p* value = 1.488e-77, strong positive association); the CGM097-*ZWILCH* pair showed a moderate correlation (ρ = 0.403, *p* value = 5.396e-19); and the CGM097-*EXD3* pair exhibited a weak correlation (ρ = 0.109, *p* value = 0.019; Figure 1B). These association patterns illustrate the differential strength of correlation between a single drug and distinct genes. Extending this analysis to all drug-gene pairs across the dataset, we generated 127,601,865 associations. Among these initial associations, the subset with a correlation coefficient greater than 0.4 corresponds to the top 3% when ranked by their correlation coefficients (Figure 1C). At the individual drug level, for alpelisib, the distribution of drug-gene association scores is consistent with the overall pattern (Figure 1D). This analysis helps effectively eliminate interference from weak associations and allows us to identify more reliable drug-gene association pairs. Based on a correlation threshold of 0.4 and a statistical significance level of 0.05, we identified 21,928 significant predicted drug-gene inhibitory associations (Table S1). Here, a positive correlation suggests that drugs may exert inhibitory effects on the corresponding genes or their pathways, as genes with high essentiality in sensitive cell lines indicate potential dependence for cell survival.

For the drug-gene pairs, correlation does not indicate direct targeting, but the positive correlation provides a basis for hypothesizing inhibitory relationships. For these associations, the total 1,963 involved genes were significantly enriched in cancer-related pathways (KEGG), including EGFR tyrosine kinase inhibitor resistance, prostate cancer, acute myeloid leukemia, and other cancer-associated pathways (Figure 1E). This result implies that the drug-associated genes may help understand their anti-cancer mechanisms.

To further assess the predicted drug-gene inhibitory associations, we utilized the LINCS L1000 dataset^23^ and performed differentially expressed genes (DEGs) analysis with drug perturbation, where 137 drug-gene pairs were matched, and these involved genes were identified as significantly differentially expressed, 98 of which exhibited significant gene downregulation (Table S2). Among these 137 matched pairs, we observed a weak negative correlation between association strength and gene expression change (ρ = −0.224, *p* value = 0.0076; Figure 1F). Furthermore, the distribution of gene expression changes in the high-correlation group was more consistent with inhibitory expectations (*p* value = 0.017; Figure 1G). Notably, some drug-gene pairs showed elevated gene expression in the L1000 transcriptional profile, yet this does not contradict the proposed inhibitory relationship. It may reflect cellular compensatory feedback upregulation in response to suppressed target activity. For instance, AMG-232 (among our 137 pairs matching the L1000) acts by binding MDM2 to block *p53* degradation, not downregulating its expression, and cells may modestly upregulate *MDM2* mRNA as compensatory feedback.^24^ This aligns with the dominant mode of action of certain anti-tumor agents, which relies on target function inhibition rather than simply gene expression downregulation.^25^ Collectively, our correlation-based approach provides a set of predicted drug-gene inhibitory associations for further investigation.

### The WEKE model and validating its independent-test performance

**Figure 2:**
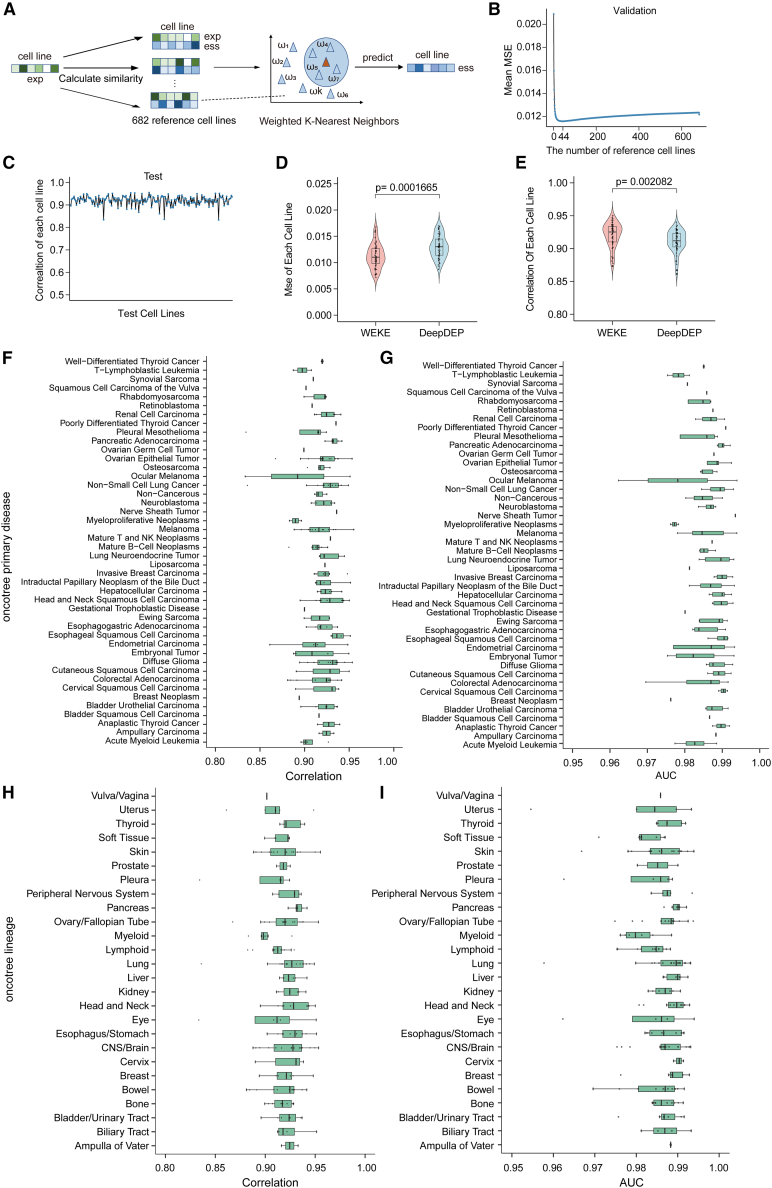
Overview and performance characterization of the WEKE model (A) Model architecture. The WEKE model leverages a weighted k-nearest neighbor procedure to predict gene essentiality in target cancer cell lines: (1) Pearson correlation, calculated based on gene expression rankings, quantifies the similarity in expression patterns between target and reference cell lines; (2) the top k most similar reference cell lines are selected; (3) gene essentiality in the target cell line is inferred via a weighted average of gene essentialities from these k reference cell lines, with weights assigned as the corresponding correlation coefficients. (B) Performance on the validation subset. MSE plotted against the number of reference cell lines. (C) Performance on the testing subset. Correlation between predicted and actual gene essentialities across all tested cell lines. (D and E) Performance comparison between WEKE and DeepDEP across 52 human cancer cell lines. These 52 cell lines are the intersection of the 204 test cell lines of our model and the feature set of DeepDEP without missing values. (F–I) Boxplots displaying Pearson correlation coefficients and AUC values of the WEKE model across 44 cancer types and 26 tissue types, respectively. For all boxplots, boxes indicate the interquartile range (IQR), center lines denote medians, and whiskers extend to 1.5× IQR. Data are represented as mean ± SD.

The essentiality profile of genes is cancer type and cancer cell line specific.^20^ In practical application, the gene essentiality of anonymous cell lines remains unclear. To address this gap, we developed WEKE, a weighted K-nearest neighbor-based tool for predicting gene essentialities in cancer cell lines. By quantifying the similarity of expression patterns between target and reference cell lines to assign distinct weights, and combining this with weighted inference using known gene essentiality data from reference cell lines, WEKE can achieve precise prediction of gene essentiality in cancer cell lines (Figure 2A).

5-fold evaluation showed that the average mean squared error (MSE) across validation folds was minimized when k was set to 44, indicating optimal model performance with weighted inference using the 44 cell lines with the most similar expression patterns as references (Figure 2B). At this parameter setting, the correlation coefficients of the test set at the cell line level fall within the range of 0.8269–0.9513 (Figure 2C). Compared to the existing DeepDEP model,^19^ our model achieved a 0.17% reduction in MSE (*t* test, *p* value = 0.0002) and a 0.98% increase in correlation coefficient (Wilcoxon rank-sum test, *p* value = 0.0021) across 52 cell lines (Figures 2D and 2E). Furthermore, in the test set, the WEKE model achieved correlation ranges of 0.8895–0.9363 across 44 disease levels and 0.8951–0.93 across 26 tissue levels. The corresponding area under the curve (AUC) value ranges were 0.9762–0.9935 and 0.9804–0.9903, respectively. Each disease or tissue level corresponds to multiple cell lines, and the ranges were calculated after taking the mean values of these cell lines (Figures 2F–2I). Additionally, the model shows distinct performance advantages in multi-task scenarios, demonstrating robust regression and classification capabilities on both the test set and the full dataset (Figures S1A–S1F). Although our improvement over DeepDep is slight, it requires much fewer input feature types (only expression profile) and can be easily applied in a practical case, for example, here determining the functional essentiality of genes directly or indirectly by a drug.

### The DeepEEAA model accurately predicts drug sensitivity and outperforms existing benchmarks

**Figure 3:**
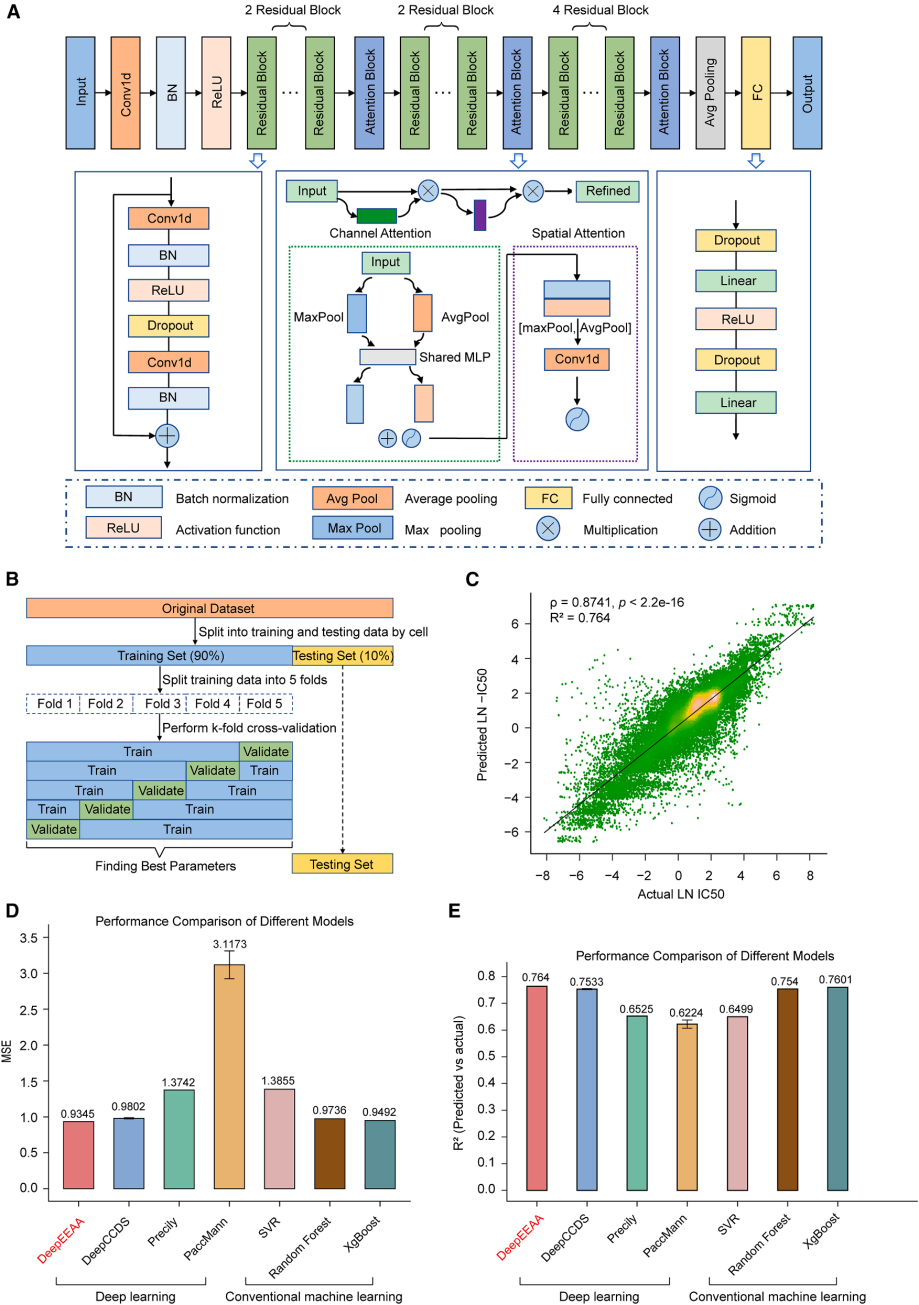
Workflow and evaluation for DeepEEAA predictive analysis (A) Schematic overview of DeepEEAA. Input features (20 dimensions) comprise gene expression, gene essentiality, drug-protein affinity, and predicted drug-gene associations, derived from 5 genes selected by descending drug-protein affinity. (B) Dataset partitioning process. The dataset was partitioned based on cell lines. (C) Scatterplot showing the performance of DeepEEAA across all cell line-drug pairs in newly fitted test data. *p values* were calculated using a two-sided *t* test. (D and E) Comparison of drug response predictions across different methods. Bar charts display the distribution of MSE (D) and R^2^ (E) values between predicted and observed log-transformed IC_50_ (LN IC_50_) values, comparing widely cited deep learning models (with different features) and machine learning models (with identical features). Data are represented as mean ± SD.

We constructed a multidimensional feature-based model to achieve accurate prediction of drug sensitivity in cancer cell lines. Specifically, the selected features encompass gene expression levels, gene essentiality, drug-protein affinity (derived from the DeepDTAGen model^26^), and predicted drug-gene associations. To conduct model training (Figure 3A), 405,857 drug-cancer cell line pairs and their corresponding sensitivity data were retrieved from the DepMap database, covering 1,813 drugs and 671 cell lines. All samples were divided into a training set and a test set at a 9:1 ratio, following the principle that cell lines do not overlap between the two sets. For the training set, 5-fold cross-validation was performed to optimize parameters, with MSE of the validation set used as the evaluation metric (Figure 3B). Validation of model performance showed that the trained DeepEEAA model exhibited highly accurate predictive capability on the independent test set (R^2^ = 0.764, *p* value < 2.2 × 10^-16^), indicating that it can effectively predict drug sensitivity in cancer cell lines (Figure 3C). Across 25 cancer types, Pearson correlation coefficients ranged from 0.825 to 0.909 (Table S3), indicating that predictive accuracy was consistent across diverse cancer lineages. We further validated DeepEEAA using datasets from the CCLE and GDSC databases, and the model maintained high predictive accuracy (Figure S2).

Subsequently, we conducted benchmarking tests to compare DeepEEAA with several widely cited recent frameworks^15^^,^^16^^,^^17^ and three traditional algorithms using identical features. Results showed that traditional machine learning algorithms also performed well with our multidimensional features. DeepEEAA achieved improved predictive performance, exhibiting higher R^2^ values and lower MSE compared with both existing deep learning frameworks and traditional machine learning algorithms (Figures 3D and 3E; Table S4). These results demonstrate the effectiveness of our multidimensional feature system while highlighting the complementary value of DeepEEAA’s deep learning architecture. Removal of the drug-gene association feature resulted in a decrease in R^2^ from 0.764 to 0.735 and a minor ranking shift (Figure S3). The main conclusions remained consistent.

### Development of the DrGee online tool for drug prioritization in cell lines using only gene expression profiles

To facilitate practical application, we developed the DrGee online tool. First, it processes user-provided gene expression data of a cell line via the computational model (WEKE) to complete gene essentiality prediction. Then, it utilizes the DeepEEAA model to conduct quantitative sensitivity analysis on the candidate drugs and outputs up to five drugs sorted by their predicted half maximum inhibitory concentration (IC_50_) values in ascending order. Additionally, the tool provides LOEUF^27^ and Shet^28^ values for the target genes of the prioritized drugs.

To validate the independence and accuracy of the DrGee tool, we selected the lung cancer cell line 95-D, which is not recorded in the DepMap database for validation. First, we obtained the gene expression levels of the 95-D cell line through RNA-sequencing (Table S5). Subsequently, based on the DrGee online tool, four drugs were output for this cell line. These drugs were predicted to be linked to essential genes, including *ZBTB8OS* (BI-97C1), *DHFR* (trimetrexate), *PSMB2* and *PSMB5* (carfilzomib), and *CHEK1* (LY2606368), indicating potential predictive inhibitory associations (Refer to Table S6 for all genes’ essentialities in the 95D cell). Based on the population genetic profiles of these genes, inhibition of *ZBTB8OS*, *DHFR*, and *CHEK1* is associated with relatively lower population-level genetic constraint, suggesting favorable safety characteristics in the context of drug targeting (Figure S4). Subsequent *in vitro* and *in vivo* experiments further supported the anticancer activity and tolerability of BI-97C1 and trimetrexate.

To explore the predicted drug-gene associative relationship identified by DrGee, we performed transcriptome sequencing on cell lines before and after BI-97C1 treatment. Results displayed significant downregulation of *ZBTB8OS* (logFoldChange = −0.779, adjusted *p* value <0.001; Figure S5), which is consistent with the predicted association between BI-97C1 and *ZBTB8OS*, suggesting a potential transcriptional response to drug treatment.

### *In vitro* validation of candidate drugs for the 95-D lung cancer cell line

**Figure 4:**
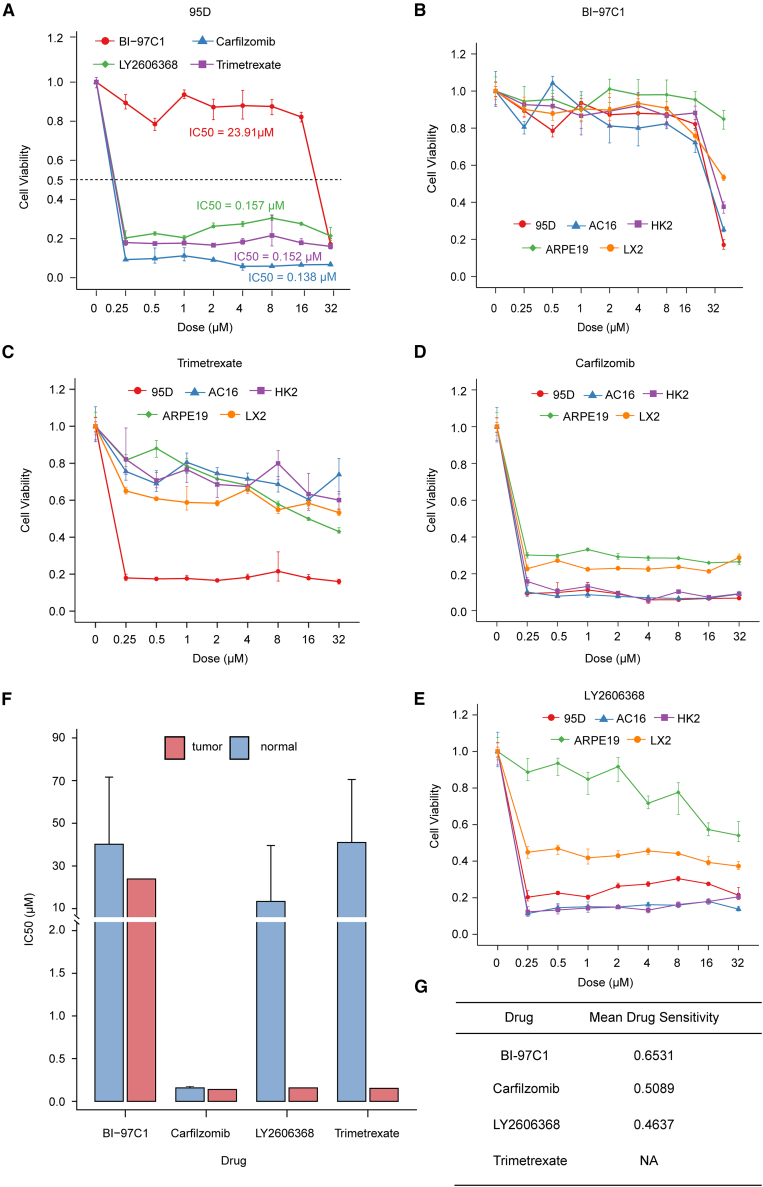
*In vitro* validation of four identified drugs in the 95-D human lung cancer cell line (A) *In vitro* validation of the candidate compounds in the 95-D lung cancer cell line. Data are represented as mean ± SD (*n* = 4). (B–E) Line plots showing cell viability of BI-97C1, trimetrexate, carfilzomib, and LY2606368 in the 95-D lung cancer cell line and the four normal cell lines (AC16, HK2, LX2, and ARPE19). Data are represented as mean ± SD (*n* = 4). (F) Bar plot of drug IC_50_ values in normal cell lines (mean ± SD, *n* = 4) and lung cancer cell line. This plot shows IC_50_ of four drugs (BI-97C1, carfilzomib, LY2605308, trimetrexate) in 95-D lung cancer (tumor, red bars) and the aforementioned normal cell lines (blue bars). A higher IC_50_ means lower drug sensitivity. (G) Table presenting potential compounds identified via the DrGee online tool in the newly sequenced 95-D human lung cancer cell line, with corresponding mean drug sensitivity AUC (from the DepMap database) and *in vitro* experimentally measured IC_50_ values.

To experimentally validate the candidate drugs identified for the 95-D lung cancer cell line by the DrGee online tool, we performed *in vitro* growth inhibition assays and determined their IC_50_ values. The experimentally determined IC_50_ values of the four candidate drugs in 95-D cells validated the predictive accuracy of DrGee for drug sensitivity estimation (Figure 4A; Table S7). To further assess the cytotoxic profiles of these compounds, we not only evaluated their effects based on target gene characteristics but also assessed their cytotoxicity at the cellular level. Four normal human cell lines representing major organs were selected for testing (Figures 4B–4E; Tables S8 and S11). BI-97C1 and trimetrexate exhibited significantly higher IC_50_ values in normal cells compared with 95-D cells, indicating relatively low cytotoxicity toward non-malignant cells. In contrast, carfilzomib and LY2606368 showed relatively low IC_50_ values in normal cell lines, suggesting stronger cytotoxic effects on non-malignant cells (Figure 4F).

To quantitatively evaluate compound cytotoxicity across different cell types, we calculated the average AUC of the drug across all tested cell lines, with lower AUC values indicating higher cytotoxicity. Carfilzomib and LY2606368 displayed average AUC values below 0.6, whereas BI-97C1 and trimetrexate exhibited average AUC values above 0.6 (Figure 4G), consistent with their lower cytotoxicity toward normal cells. Based on these results, an average AUC threshold of 0.6 was defined as a cytotoxicity-based exclusion criterion for the *in vitro* screening, and drugs with average AUC values below this threshold were excluded. Incorporation of this criterion into the DrGee workflow ultimately identified BI-97C1 and trimetrexate as the most suitable candidates for further investigation for the 95-D lung cancer cell line.

### *In vivo* validation of candidate drugs for the 95-D lung cancer cell line in a mouse xenograft model

**Figure 5:**
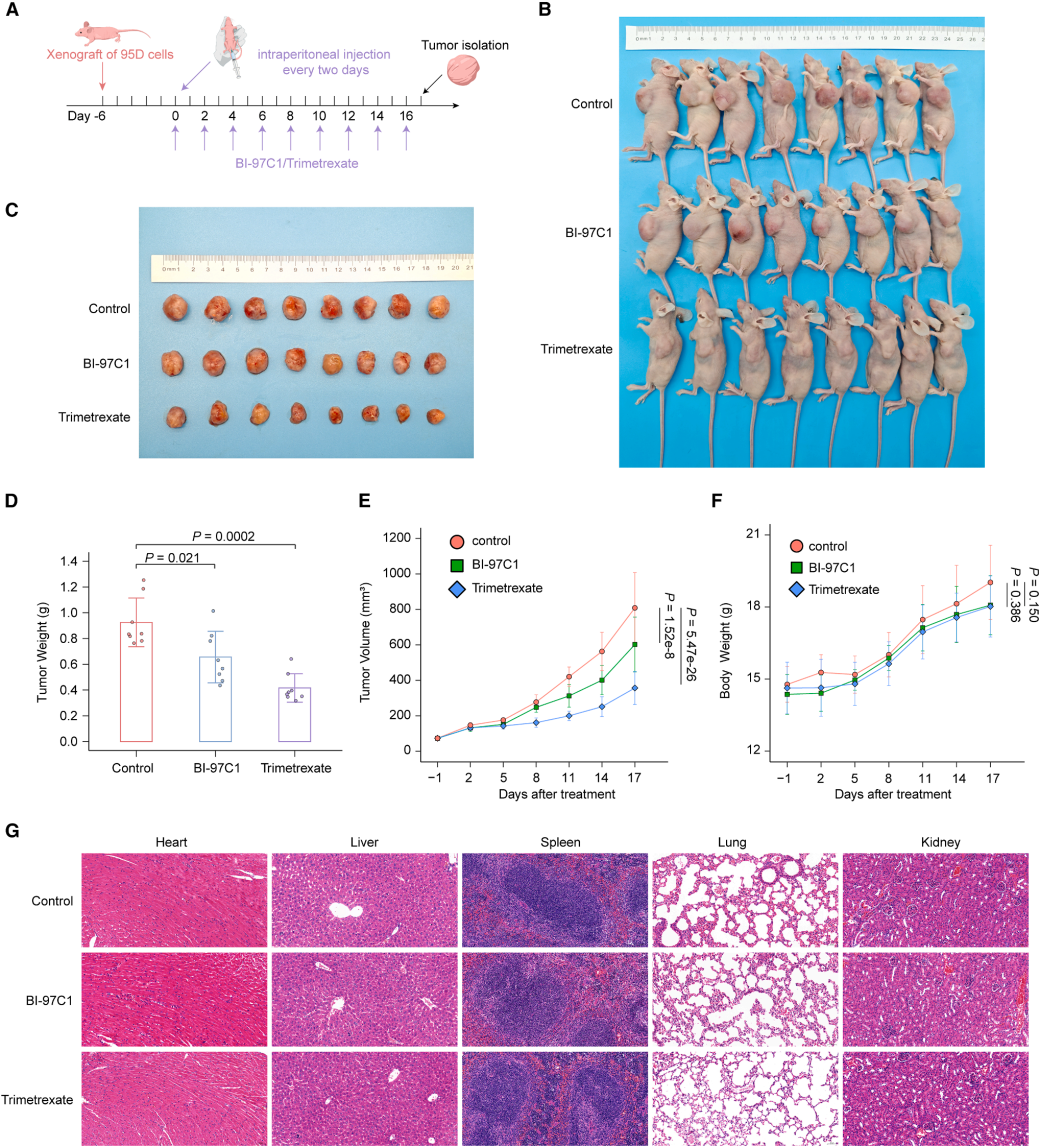
Validation of the drug identifying workflow using a mouse lung cancer model (A) Schematic of the *in vivo* experimental design. Mice bearing 95-D lung cancer cells received intraperitoneal injections of saline (control), BI-97C1 (5 mg/kg), or trimetrexate (60 mg/kg). Drug administration was initiated when the tumor volume reached 100 mm^3^; arrows indicate drug injection time points. (B) Images showing the overall appearance of mice bearing 95-D lung cancer cells after completion of treatment. (C) *Ex vivo* images of tumor tissues from each group at the end of treatment. (D) Tumor weight at the end of treatment. (E) Average tumor growth curve during treatment. (F) Changes in mouse body weight during treatment. Data in D–F are presented as mean ± SD (*n* = 8; two-way ANOVA followed by Bonferroni multiple-comparison post-test). (G) Histological observation of major organs in 95-D mice-bearing mice at the end of treatments by H&E staining. Scale bar: 50 μm.

To assess the *in vivo* efficacy of the candidate agents, we established a 95-D lung cancer xenograft model and defined the optimal intervention doses for both compounds via preliminary experiments (Figures S6A–S6C; Tables S12 and S13). Drug administration was initiated in 95-D tumor-bearing mice: BI-97C1 or trimetrexate was delivered by intraperitoneal injection every 2 days, for a total of 9 doses. Throughout the experiment, tumor volume and mouse body weight were measured every 3 days, and the overall condition and food intake of the mice were continuously monitored (Figure 5A). Compared with the control group, both BI-97C1 and trimetrexate treatments significantly suppressed the growth of xenograft tumors, as evidenced by marked reductions in tumor volume and weight. Specifically, tumor volume was decreased by 25.6% and 55.8% in the BI-97C1 and trimetrexate groups, respectively. These findings demonstrate that both agents exert significant *in vivo* antitumor activity in a lung cancer xenograft model (Figures 5B–5E; Tables S14 and S15). No overt adverse effects of BI-97C1 and trimetrexate were observed, as evidenced by body weight measurements and H&E staining of major organs. (Figures 5F and 5G).

### Validation of the DrGee tool in ovarian cancer

To further evaluate the utility of this study’s methodology, we used the OVCAR8 human ovarian cancer cell line as the research subject. Using the DrGee online tool, we predicted and output five candidate compounds: digoxin, TG-02, mevastatin, pitavastatin, and selinexor. Simultaneously, the tool outputs the predicted gene inhibition information and key characteristics of these drugs (Table 1). Furthermore, the sensitivity of this cell line to these drugs was also examined using the DepMap database (Figure S7). Notably, although pitavastatin is a commonly used non-oncological drug primarily for treating hypercholesterolemia, several studies have suggested it exerts potential antitumor effects.^29^^,^^30^ In this study, predictions from the DrGee tool further support this possibility. Collectively, these findings provide support for the potential effectiveness of the DrGee tool in the cancer cell lines examined.

**Table 1:** Drug ranking for OVCAR8 cell line generated by DrGee

Drug	Prediction (LN IC_50_)	Mean drug Sensitivity	Gene	Gene essentiality	LOEUF	S_het_
PITAVASTATIN	−4.48	0.8079	*HMGCR*	0.9725	0.447	0.1338
SELINEXOR	−3.543	0.6108	*XPO1*	0.9964	0.083	NA
DIGOXIN	−2.519	0.6582	*ATP1A1*	0.8558	0.188	0.3546
TG-02	−2.184	0.7803	*CDK9*	0.9925	1.139	0.01861
MEVASTATIN	−2.107	0.8245	*HMGCR*	0.9725	0.447	0.1338

### TCGA clinical response-based model confirmed the effectiveness of essentiality-centered features

**Figure 6:**
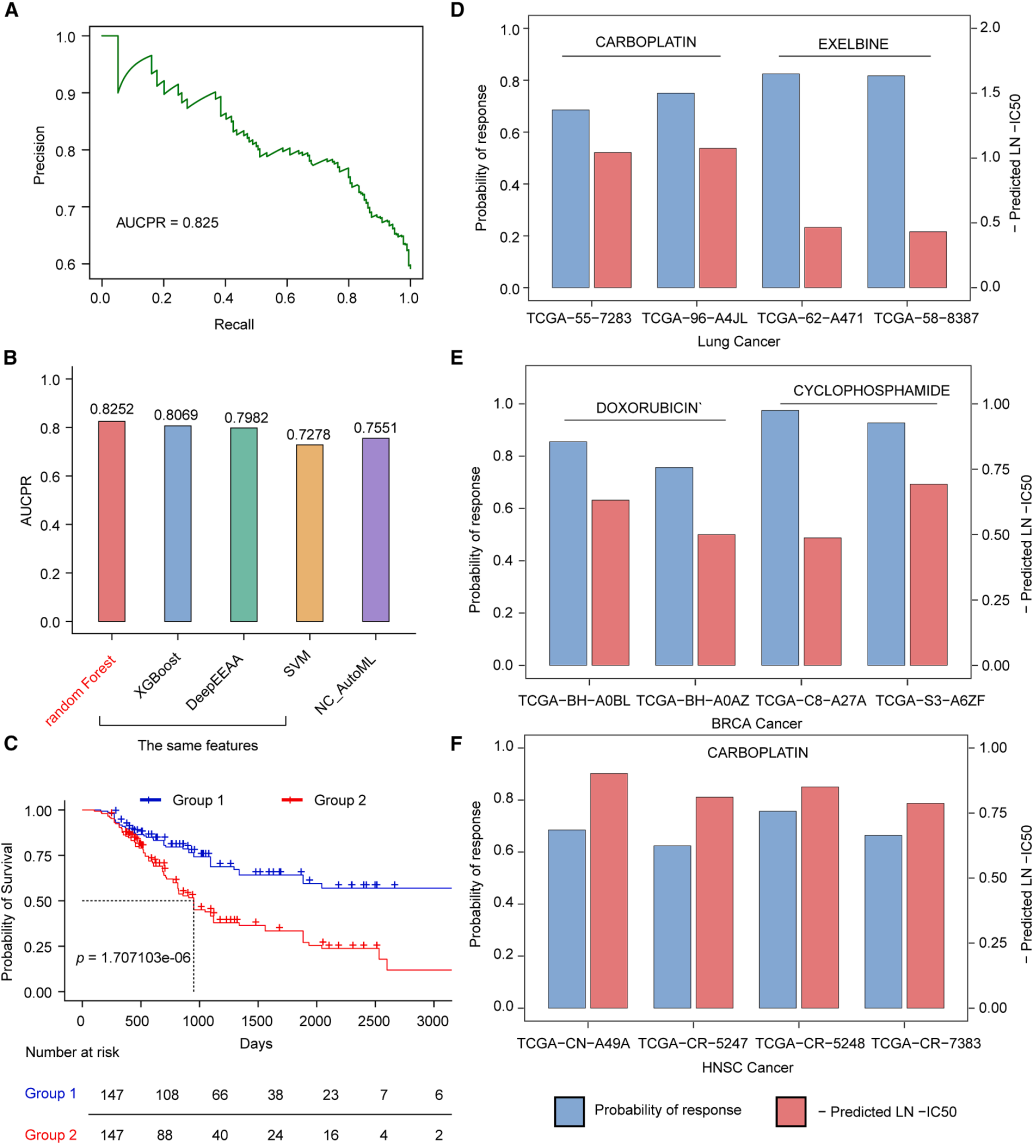
Evaluation of TCGA drug-patient response model (A) Precision-recall curve showing the performance of the best random forest model on the TCGA drug-patient response test dataset. (B) Comparison of AUC-PR across different models on the same TCGA test dataset. The first four models were trained using our previously described features, and the last model is the one proposed by Chawla et al.^16^ (C) Kaplan-Meier survival analysis across multiple cancer types in the TCGA dataset. Patients were stratified into two groups based on the median predicted probability of response. Survival differences were assessed using the log rank test. (D–F) Bar plots showing predicted response probability (left *y* axis) and negative predicted IC_50_ values (right *y* axis) for drugs indicated above the plots in lung cancer (D), breast cancer (BRCA; E), and head and neck squamous cell carcinoma (HNSC; F), respectively. Blue bars represent response probability generated by the random forest model, and red bars represent negative predicted IC_50_ values generated by the DeepEEAA model.

TCGA^31^ encompasses gene expression profiles across multiple cancer types and patients’ clinical data, as well as drug response information. We trained models using the four previously described essentiality-centered features, among which the random forest model achieved an area under the precision-recall curve (AUC-PR) of 0.825 on the test set, showing improved performance compared with the previously reported AutoML model^16^ (Figures 6A and 6B). Independently, to evaluate whether predicted drug response probabilities can serve as a survival risk stratification metric, we stratified patients into two groups for survival analysis with the median predicted response probability as the cutoff. Notably, patients predicted to have a positive drug response by the model exhibited a significantly improved overall survival rate (*p* value [log rank test] < 0.001; Figure 6C). Multivariate Cox regression analysis incorporating predicted drug response probability along with clinical covariates (cancer type, age, sex, and tumor stage) showed that the predicted probability was independently associated with overall survival (adjusted HR = 0.20, 95% CI: 0.08–0.50, *p* < 0.001; C-index = 0.755; Figure S8).

Further, we evaluated the coincidence between outputs of the clinical response and IC_50_ prediction models across distinct cancer types and their corresponding chemotherapy regimens. The selected agents were all clinically confirmed positive-response drugs. Random forest-derived response probabilities and DeepEEAA-derived LN-IC_50_ values exhibited a consistent trend across TCGA samples. Specifically, higher predicted probabilities were associated with lower LN-IC_50_ values for carboplatin/paclitaxel in lung adenocarcinoma (LUAD), doxorubicin/cyclophosphamide in breast cancer (BRCA), and carboplatin in head and neck squamous cell carcinoma (HNSC) (Figures 6D–6F). Collectively, these results indicate that models trained on essentiality-centered features can yield predictions of drug sensitivity and treatment response that show consistency across the cancer types and regimens examined, suggesting the potential utility of these features.

## Discussion

Gene essentiality represents a fundamental determinant of cancer cell survival and therapeutic vulnerability. Variability in gene essentiality not only influences cancer cell sensitivity to pharmacological perturbations but also exhibits pronounced heterogeneity across cancer types and cellular contexts.^32^^,^^33^^,^^34^ Compared with non-essential genes, essential genes represent core survival dependencies that are less affected by stochastic or non-specific transcriptional variation, thereby providing a more functionally grounded representation of cancer cell vulnerability to drug intervention.^35^^,^^36^^,^^37^ From a translational perspective, essentiality captures functional dependencies that may not be apparent from genomic alterations alone. In this study, we propose an essentiality-centered framework for drug sensitivity prediction and prioritization, positioning gene essentiality as a key biological axis linking gene expression, drug-gene associations, and drug response.

The proposed framework comprises three complementary components. First, we systematically characterized drug-gene inhibitory associations by correlating gene essentiality profiles with drug sensitivity profiles across large-scale cancer cell line datasets. This strategy anchors drug response prediction to functional gene dependencies rather than relying solely on molecular annotations or chemical similarity. Second, we demonstrate that gene essentiality can be inferred directly from gene expression profiles, enabling applicability in settings where comprehensive genomic profiling is unavailable. Third, drug sensitivity is quantitatively inferred by integrating predicted gene essentiality, drug-protein affinity, and the strength of gene–drug associations. Together, these components constitute the DrGee online platform, which enables systematic ranking of candidate drugs using only gene expression data, with prioritized compounds corresponding to lower predicted IC_50_ values. Notably, the framework does not depend on extensive multi-omics inputs such as somatic mutations, copy number alterations, or DNA methylation, thereby enhancing its feasibility across diverse experimental and clinical contexts.^15^^,^^16^^,^^17^^,^^18^^,^^22^^,^^38^

Experimental validation in the highly metastatic 95-D lung cancer cell line provides preliminary support for the potential efficacy of this framework. Drugs prioritized by the DrGee platform, including BI-97C1 and trimetrexate, exhibited pronounced anti-proliferative effects in cancer cells while maintaining relatively low cytotoxicity in normal cell lines. BI-97C1 (sabutoclax) was originally developed as a pan-Bcl-2 family inhibitor and has shown preclinical antitumor activity in multiple malignancies, including prostate cancer, lymphoma, and lung cancer models.^39^ Trimetrexate, a lipophilic dihydrofolate reductase inhibitor clinically used for *Pneumocystis* pneumonia, has also been explored for its anticancer potential in solid tumors, including non-small cell lung cancer.^40^ Transcriptomic profiling following drug treatment further corroborated the predicted drug-gene associations, as exemplified by the downregulation of *ZBTB8OS* after BI-97C1 exposure. Importantly, *in vivo* xenograft experiments demonstrated significant tumor growth inhibition with no overt adverse effects observed, suggesting that essentiality-guided drug prioritization can identify candidates with favorable efficacy and tolerability profiles in this experimental setting. Collectively, these findings indicate that, although the predictive model operates at a computational level, it is built upon biologically informed features, including gene essentiality, drug protein affinities, and gene-drug associations, supporting the potential utility of this feature-based framework for drug prioritization.

Compared with existing drug response prediction approaches, the present framework has several potential methodological advantages in contexts where multi-omic data are not readily available. First, it requires minimal input data, relying exclusively on gene expression profiles, which are commonly obtainable in both experimental and clinical settings. Second, by explicitly modeling the collective contribution of drug-inhibited genes rather than focusing on a limited set of canonical targets, the framework is designed to account for polypharmacological dependencies that are increasingly recognized as central to anticancer drug efficacy.^41^^,^^42^^,^^43^ This essentiality-centered, multi-target design enables quantitative inference of drug sensitivity and shows favorable predictive accuracy in our benchmarking analyses, with lower MSE than several baseline methods in independent testing^15^^,^^16^^,^^17^ (Table S4). Third, the DrGee platform encompasses 4,935 compounds, including approved anticancer agents and investigational drugs with experimentally validated IC_50_ values, providing a scalable resource to support systematic drug repurposing and exploration of therapeutic options in cancer contexts lacking established targeted treatments. Notably, drug prioritization is directly conditioned on the input gene expression profile, allowing the framework to capture both cell line-specific and shared drug sensitivities across different cellular contexts. Nevertheless, the drug-gene association feature was computed using the full dataset to ensure statistical stability, which could potentially raise a concern about information leakage. To assess this, we performed an ablation study by completely removing this feature and retraining the model. The minor decrease in R^2^ (from 0.764 to 0.735) confirms that this feature does not substantially influence the predictions, indicating that any potential leakage, if present, does not materially affect the reported results or conclusions.

Beyond preclinical applications, we explored whether essentiality-centered features could be relevant in clinical contexts by leveraging TCGA patient drug response data. Using TCGA patient drug response data,^16^^,^^31^ we trained a random forest classification model based on the same feature space employed for cell line-level prediction. This model achieved promising performance, with an AUC-PR of 0.825, and identified patient subgroups with significantly different overall survival outcomes, suggesting a potential association between the predicted probabilities and clinical prognosis. Moreover, predicted response probabilities showed a consistent trend with regression-based drug sensitivity estimates derived from the DeepEEAA model, with higher predicted response probabilities associated with lower predicted LN-IC_50_ values across multiple cancer types and therapeutic regimens. Although these retrospective findings are preliminary, prospective validation will be required to establish whether essentiality-based predictions have potential utility for patient stratification or exploratory treatment prioritization.

In summary, this study presents an essentiality-centered framework that requires only gene expression data as input for precision oncology drug prioritization, integrating predicted gene essentiality with drug-target and gene-drug association information. At the preclinical level, the DrGee platform enables quantitative prediction of drug sensitivity and systematic prioritization of candidate compounds for individual cancer cell lines. Preliminary analyses further suggest that essentiality-based features may support patient stratification in retrospective datasets, with exploratory analyses revealing associations with differences in survival outcomes. By positioning gene essentiality as a central organizing principle linking molecular profiles to drug response, this framework offers a potentially scalable approach for drug prioritization based on functional dependency features and provides a foundation for rational exploration of both oncologic and non-oncologic therapeutic options.

### Limitations of the study

Several limitations of this study should be acknowledged. First, our safety assessment combines population-level gene constraint metrics, including LOEUF^27^ and Shet^28^ values, with *in vitro* cytotoxicity measurements based on average AUC values in normal cell lines. These measures together serve as pragmatic indicators for initial safety screening. However, they remain indirect proxies and do not capture important pharmacological factors, such as tissue specificity, pharmacokinetics, or off-target effects. Therefore, while this approach is useful for preliminary safety ranking, it does not constitute a comprehensive toxicity assessment. Future efforts should focus on establishing predictive models that integrate multi-dimensional pharmacological data to better inform clinical safety evaluation.^44^^,^^45^ Second, the DeepEEAA model was trained and evaluated using cell-line-wise dataset partitioning rather than drug-wise splitting. While this strategy aligns with our primary objective of predicting drug responses for existing drugs in new biological contexts, it does not assess model generalization to entirely novel compounds (Figure S9). Notably, this limitation reflects a broader challenge in the field, as evaluating predictive performance on unseen compounds remains a common difficulty in drug response modeling.^46^^,^^47^^,^^48^

Additionally, this model is not interpretable despite biologically grounded inputs; future work will focus on interpretable architectures to better align predictions with biology. Third, the scope of empirical validation is limited. Our own experimental validation was performed in only two representative cell lines (95-D and OVCAR8), broader validation across additional cancer models and drug classes is deferred to future studies. Separately, the clinical analyses in this study were performed using the TCGA drug response dataset, which has limited sample sizes and heterogeneous treatment contexts across cancer types. We used this dataset to maximize available data for exploratory model development. These findings are therefore intended to illustrate the potential of essentiality-based features for patient stratification rather than to establish definitive clinical utility, and prospective validation in larger independent cohorts is warranted. Although models for predicting patient-specific drug responses were developed, this functionality is not currently integrated into the DrGee platform. Finally, prioritized drugs may not exert antitumor effects primarily through inhibition of the predicted essential genes. Drug-gene associations inform sensitivity prediction but do not imply confirmed causal mechanisms. Inferred essential genes should therefore be interpreted as predictive features rather than definitive therapeutic targets, underscoring the continued need for experimental validation.

## Resource availability

### Lead contact

Further information and requests for resources and reagents should be directed to and will be fulfilled by the lead contact, Feng-Biao Guo (fbguoy@whu.edu.cn).

### Materials availability

This study did not generate new unique reagents.

### Data and code availability

The original data were obtained from the DepMap: https://depmap.org/portal/. Processed results generated in this study are available in the DrGee: http://gepa.org.cn/DrGee with unrestricted access. All source codes used in this study are publicly available in the GitHub: https://github.com/hongtucui/DrGee and permanently archived at Zenodo: https://doi.org/10.5281/zenodo.21286516. In addition, all raw RNA-seq data generated in this study have been deposited in NCBI BioProject: PRJNA1482717 and will be publicly available upon publication. No access restrictions are applied to these datasets.

## Acknowledgments

This work was supported by the 10.13039/501100001809National Natural Science Foundation of China (grant no. 32370696), the Climbing Project for Medical Talent of Zhongnan Hospital of Wuhan University (grant no. PDJH202406), Noncommunicable Chronic Diseases-National Science and Technology Major Project (grant no. 2023ZD0506200/2023ZD0506202).

## Author contributions

F.-B.G. conceived the study, guided the design of the models, and took part in revising the manuscript. J.L., Z.C., and F.-B.G. supervised the project. H.C. designed and fully implemented the machine learning model and online tool, performed data analysis, conducted *in vitro* experiments, and drafted the manuscript. X.D. contributed to experimental work and data analysis. H.G., Z.L., J.F., J.L., and Z.C. took part in revising the manuscript. H.C., B.W., J.-Y.Z., and X.L. performed the data collection and preprocessing. A.Y., X.L., and D.Z. checked the results. All authors contributed to the discussion and revision of the final version.

## Declaration of interests

The authors have declared no competing interests.

## STAR★Methods

### Key resources table

**Table:** 

REAGENT or RESOURCE	SOURCE	IDENTIFIER
**Chemicals, peptides, and recombinant proteins**		

BI-97C1	Bide Pharmatech	Cat# BD764862
LY2606368	Bide Pharmatech	Cat# BD566146
Carfilzomib	Yuanye Bio-Technology	Cat# S51335
Trimetrexate	Yuanye Bio-Technology	Cat# S85635
Trizol(Total RNA Extraction Kit)	Yuanye Bio-Technology	Cat# R21086
Enhanced CCK-8 Solution	MeilunBio	Cat# MA0218

**Critical commercial assays**		

Eukaryotic transcriptome sequencing	Berry	N/A

**Deposited data**		

DepMap	DepMap (version 23Q4	DepMap: https://depmap.org/portal/
DrugBank	DrugBank 5.0	DrugBank: https://go.drugbank.com/
ClinicalTrials	N/A	ClinicalTrials: https://clinicaltrials.gov/
LINCS L1000 dataset	Level 3	LINCS L1000: https://clue.io/releases/data-dashboard
gnomAD	gnomAD v4.1.1	gnomAD: https://gnomad.broadinstitute.org/
RGC Million Exome Variant Browser	N/A	RGC Million Exome Variant Browser: https://rgc-research.regeneron.com/me/license-and-terms-of-use
Raw sequencing reads	This paper	BioProject: PRJNA1482717
Code for model development	This paper	GitHub: https://github.com/hongtucui/DrGee, https://doi.org/10.5281/zenodo.21286516
Website for DrGee	This paper	DrGee: http://gepa.org.cn/DrGee

**Experimental models: Cell lines**		

95-D	Wuhan Saikangte Company	Cat# CHCH-0171
AC16	Wuhan Saikangte Company	Cat# CHCH-0265
HK-2	Wuhan Saikangte Company	Cat# CHCH-0020
LX-2	Wuhan Saikangte Company	Cat# CHCH-0160
ARPE-19	Wuhan Saikangte Company	Cat# CHCH-0283

**Experimental models: Organisms/strains**		

Mouse: BALB/c nude mice	Shouzheng Pharma (Wuhan) Biotechnology	N/A

**Software and algorithms**		

Python	Version 3.9.16	https://www.python.org/
PyTorch	Version 2.6.0	https://pytorch.org/
Numpy	Version 2.0.1	https://numpy.org/
Pandas	Version 2.2.3	https://pandas.pydata.org/
Hyperopt	Version 0.2.7	http://hyperopt.github.io/hyperopt/
scikit-learn	Version 1.6.1	https://scikit-learn.org/
R software	Version 4.3.1	https://cran.r-project.org/
Diango	Version 3.2.23	https://www.djangoproject.com/
Fastp	Version 0.23.2	https://github.com/OpenGene/fastp
Hisat2	Version 2.2.1	https://github.com/DaehwanKimLab/hisat2
Samtools	Version 1.20	http://samtools.sourceforge.net
featureCounts	Version 2.0.6	http://www.bioconductor.org
DESeq2	Version 1.40.2	http://www.bioconductor.org/packages/
Django	Version 4.2.3	https://www.djangoproject.com

### Experimental model and study participant details

#### Mice

Five-to six-week-old female BALB/c nude mice were acclimatized for 1 week in a specific pathogen-free animal facility to to eliminate environmental stress interference. Housing conditions strictly complied with the standards outlined in Laboratory Animal Requirements of Environment and Housing Facilities (*GB 14925–2010*), with specific parameters as follows: temperature 22°C–25 °C, relative humidity 40%–60%, 12 h light/12 h dark cycle (light period: 07:00–19:00), ventilation rate of 15–20 air changes per hour. All cages and bedding were sterilized by autoclaving, and mice had *ad libitum* access to sterile feed and drinking water.

Animal studies were conducted in accordance with ethical regulations approved by the Animal Welfare Ethics Review Committee of Shouzheng Pharma (Wuhan) Biotechnology Co., Ltd. (Approval No. 2025092801). All animal experimental procedures were reported following the ARRIVE guidelines.

To establish the 95-D lung cancer xenograft model, logarithmically growing 95-D cells were prepared as single-cell suspensions. A total of 1 × 10^7^ 95-D cells suspended in 0.1 mL serum-free medium were subcutaneously injected into the left axillary fossa of each nude mouse. Tumor growth was dynamically monitored post-inoculation, and drug treatment was initiated when the maximum tumor volume reached approximately 100 mm^3^.

#### Cell lines

The human lung cancer cell line 95-D (Cat# CHCH-0171) was purchased from Wuhan Saikangte Company and cultured in RPMI 1640 medium supplemented with 10% fetal bovine serum (FBS). The human cardiomyocyte line AC16 (Cat# CHCH-0265), human proximal renal tubule cell line HK-2 (Cat# CHCH-0020), human hepatic stellate cell line LX-2 (Cat# CHCH-0160), and human retinal pigment epithelial cells (Cat# CHCH-0283) were also purchased from the same vendor, and cultured in DMEM supplemented with 10% FBS.

All cell lines were authenticated via short tandem repeat (STR) profiling by the vendor upon purchase, and all culture media were additionally supplemented with 1% penicillin/streptomycin (P/S) mixture to prevent microbial contamination. Cells were incubated at 37 °C with 5% CO_2_, and routine mycoplasma testing was performed throughout cell culture. Only cells in the logarithmic growth phase were harvested for subsequent experiments.

### Ethics approval and consent to participate

Animal studies were conducted in accordance with ethical regulations approved by the Animal Welfare Ethics Review Committee of Shouzheng Pharma (Wuhan) Biotechnology Co., Ltd. (Approval No. 2025092801). All methods are reported in accordance with the ARRIVE guidelines.

### Method details

#### Data collection and preprocessing

All data utilized in this study were retrieved from DepMap (version 23Q4),^14^ which comprises three datasets: (1) CRISPR-related datasets: the CRISPR gene knockout dataset, which covers 18,424 genes across 1,100 cell lines; and the CRISPR gene essentiality dataset, which encompasses 18,443 genes across 1,100 cell lines; (2) the gene expression dataset, which contains transcriptional expression levels of 19,144 genes across 1,479 cell lines; (3) the drug-cell line sensitivity dataset, which comprises drug response data curated from five sources: the Primary database, the Secondary database, the CTD2 database, and the GDSC1/2 database.

During the data preprocessing stage, we first performed deduplication on drug datasets from five sources, yielding 4,904 unique drugs and 1,241 unique cell lines (Figures S10A–S10C). Subsequently, systematic screening was conducted across three independent dimensions: (1) Matching CRISPR gene knockout data with gene expression data identified 1,100 cell lines and 18,405 genes covered by both datasets; (2) Matching drug sensitivity data with gene expression data in 961 cell lines containing both types of information (for subsequent analysis); (3) Matching gene expression data with gene essentiality data selected 18,225 genes and 1,021 cell lines with complete information in both categories. In addition, in December 2024, we screened marketed anticancer drugs from the DrugBank database.^49^ After excluding those already present in the DepMap database, we ultimately obtained 4,935 drugs. Status information for these drugs was supplemented from the DrugBank and ClinicalTrials databases (Figure S10D).

#### Correlation calculation of gene essentiality-drug sensitivity among cell lines

We calculated Pearson correlation coefficients between drugs’ sensitivities (quantified as AUC, area under the dose-response curve) and genes’ essentialities to assess the strength of their association among cell lines. Two-slided t-tests were performed to obtain the *p*-values for the correlation coefficients of each gene-drug pair. Subsequently, duplicate cell lines were removed, and we ultimately retained 682 cell lines with complete information (including expression levels, gene essentiality, and gene-drug association data). These cell lines served as the reference cell line set for subsequent analysis and modeling.

#### Construction of a gene essentiality prediction model for cancer cell lines

To predict the essentiality of each gene in specific cancer cell lines, we employed a weighted k-nearest neighbor algorithm. This approach combines known gene essentiality information from reference cell lines with weighted inference based on expression pattern similarity across cell lines. Specifically, using the R package caret,^50^ we randomly divided the 1,021 cell lines into a training set (817 cell lines, 80%) and a test set (204 cell lines, 20%), adhering to the principle that no cell line overlaps between the two sets. The formula for predicting the essentiality of each gene in the target cell line is:

$$Ess=\frac{\sum_{i=1}^{k}{corr}_{i}\;*\;{ess}_{i}}{\sum_{i=1}^{k}{corr}_{i}}$$

Here, *corrᵢ* represents the Pearson correlation between the target cell line and the *i-th* reference cell line. The specific calculation method was as follows: Genes from both cell lines were sorted by their expression levels in descending order (genes with identical expression levels are assigned the same rank). The Pearson correlation coefficient was then computed based on the respective gene rankings in both cell lines, thereby quantifying the overall similarity of their gene expression patterns. After correlations against target cell lines were achieved for all reference cell lines, the cell lines were sorted by this correlation coefficient in descending order. *Essᵢ* denotes the known essentiality of the gene in the *i-th* reference cell line, and k represents the number of reference cell lines involved in the prediction. To select the optimal hyperparameter *k*, we performed a 5-fold evaluation using the fixed reference set described above, without involving any model training. The training set was randomly divided into five non-overlapping validation subsets, containing 162, 164, 163, 164, and 164 cell lines, respectively. For each validation subset, any cell line overlapping between the validation subset and the fixed reference set was temporarily removed from the reference set. Predictions were made based solely on the remaining reference cell lines using the above algorithm, and the MSE was computed. Only the current fold was used; the other 4-folds were not used. The optimal k was determined by minimizing the average MSE across the 5-folds.

#### LOEUF and the shet population-level gene data

LOEUF quantifies a gene’s tolerance to pLoF variants. Specifically, this value reflects the intensity of negative selection acting on a gene, with 0.6 typically used as the threshold. Values below this threshold indicate strong negative selection on the gene, signifying low tolerance to pLoF variants; values above this threshold indicate higher tolerance to pLoF variants. This data was sourced from the gnomAD database.^27^ The Shet value measures heterozygous selection constraint, representing the impact of loss-of-function variants in the heterozygous state on an organism’s fitness. An average Shet value exceeding 0.073 indicates that heterozygous loss-of-function variants in such genes result in a greater reduction in organismal fitness: these variants are relatively more harmful and more likely to be associated with dominant pathogenicity. This data was obtained from the RGC Million Exome Variant Browser.^28^

#### Re-fitting of IC_50_ data for cell line drug sensitivity assessment

In studies focusing on drug sensitivity prediction, researchers typically adopt the GDSC or CTD2 databases as benchmark datasets. Nevertheless, these databases are associated with inherent limitations, including a relatively constrained quantity of drugs and cell lines, the presence of drug redundancy, and a deficiency in systematic data integration. To address these issues, this study jointly used four drug-cell line sensitivity datasets (Secondary, CTD2, GDSC1, and GDSC2) from the DepMap database. The “dr4pL” package^51^ in R software was employed to refit the dose-response curves. For duplicate drug-cell line data, median values were used for merging. The final dataset contains 1,851 drugs, 1,225 cell lines, and 560,873 drug-cell line pairs with IC_50_ values (Figures S11A and S11B).

#### Construction of a cancer drug sensitivity prediction model

Drug response prediction was formulated as a regression task. We merged the refitted IC_50_ dataset with datasets containing gene expression levels and gene essentiality data, ultimately obtaining 405,857 drug–cell line pairs. These pairs were partitioned into a 90% training set (362,676 pairs) and a 10% test set (43,181 pairs) following the principle that no cell lines overlapped between the two sets. For hyperparameter tuning, 5-fold cross-validation was employed on the training set, with the five validation subsets containing 77,522, 71,500, 73,143, 75,697, and 64,814 pairs, respectively. Input features including gene expression levels, gene essentiality, drug–protein affinity, and calculated drug-gene associations were derived from genes selected based on drug–protein affinity. For each drug, genes from calculated drug–gene associations were merged with its known target genes, sorted by descending drug–protein affinity, and the top five were prioritized. When the merged set contained fewer than five genes, the highest affinity genes were added to ensure exactly five genes per drug, standardizing the input feature dimensionality to 20 across all drugs. Using these constructed features, multiple machine learning models were implemented for quantitative drug response prediction.

For machine learning models, XGBoost, random forest, and SVR were implemented in python,^52^ with features standardized prior to training and hyperparameters optimized via grid search: XGBoost was constructed with candidate parameters including number of trees (50, 100, 200, 300, 500), maximum tree depth (3–8), and learning rate (0.01, 0.1, 0.2, 0.3); Random Forest was built with candidate parameters including number of trees (50, 100, 200, 300) and maximum tree depth (10, 20, 30, 40, 50); SVR was implemented using a radial basis function (RBF) kernel with candidate parameters including regularization parameter C (0.01, 0.1, 1, 10, 100) and kernel coefficient gamma (0.0001, 0.001, 0.01, 0.1). For each parameter combination across the three models, training and evaluation were performed using 5-fold cross-validation, with the MSE calculated for each validation fold, the optimal parameter combination selected by minimizing the average MSE, and each model was ultimately trained on the full training dataset using its respective optimal hyperparameters.

For the deep learning model, we utilized the PyTorch framework to construct and train a one-dimensional residual convolutional neural network integrated with residual connections and the Convolutional Block Attention Module.^53^ Input features were first standardized and appended with a single-channel dimension, followed by processing through an initial 3 × 3 convolutional layer (stride = 1, padding = (1) in conjunction with batch normalization and Rectified Linear Unit (ReLU) activation. The network backbone consisted of three sets of residual block layers, where each set contained 2–4 residual blocks, and the initial channel numbers were 32, 64, and 128, respectively. Notably, stride-2 downsampling was incorporated in the 2nd and 3rd sets, and a CBAM attention module (with channel compression ratios of 4, 8, and 16 for the three sets) was embedded subsequently to each set. Each residual block comprised two 3 × 3 convolutional layers, followed by batch normalization, ReLU activation, and dropout (rate: 0.1–0.5); shortcut connections were employed to ensure cross-layer dimension matching. Prior to being fed into two linear layers (with intermediate units of 64, 128, or (256) for output generation, the features were subjected to adaptive average pooling. Hyperparameter optimization was performed using the Tree-structured Parzen Estimator (TPE) algorithm implemented in Hyperopt.^54^ Optimal parameters were selected via 5-fold cross-validation based on the MSE of the validation set. The hyperparameter search space included: learning rate (10^-5^ to 10^-(2^) and minimum learning rate (10^-7^ to 10^-5^) of the Adam optimizer, reduction factors (0.1, 0.3, 0.(5) of the learning rate scheduler, and patience values (3, 5, (7) of the scheduler. During model training, early stopping (patience = 10), a batch size of 128, and a maximum of 30 epochs were adopted, with MSE serving as the loss function. A total of 500 hyperparameter evaluations were conducted to determine the optimal parameter configuration. Subsequently, the model was retrained with these optimal parameters on the full dataset for 40 epochs, followed by performance evaluation on the independent test set.

#### Construction of the DrGee online tool

We developed a user-friendly web server (DrGee), which is freely accessible at http://gepa.org.cn/DrGee. This tool enables users to input gene expression data from cell lines of interest and obtain two key outputs: predicted gene essentialities in the input cell line, and corresponding drug recommendations. All results are downloadable directly from the results page. Technically, the server was built on Python 3 with PyTorch for model construction.

#### RNA extraction, bulk RNA sequencing, and upstream data analysis for validated cell lines

For cells in the exponential growth phase, the culture medium was discarded, and the cells were briefly rinsed with 5 mL of phosphate-buffered saline (PBS), followed by discarding the rinse solution. An appropriate volume of TRIzol reagent (Biosharp) was added, and the mixture was repeatedly pipetted until the cells were completely lysed into a clear homogenate. The resulting lysate samples were submitted to Berry Genomics for library preparation and paired-end sequencing.

Raw RNA sequencing data were processed using a custom workflow: Fastp^55^ was used for quality control and trimming of paired-end sequencing files. This step included automatic adapter removal, trimming of 10 bp from the 5′ end, and sliding-window filtering of low-quality sequences to generate clean reads. Next, HISAT2^56^ was employed to align the clean reads to the transcript index of the reference genome, producing alignment results in SAM format. These SAM files were then converted to sorted BAM files and indexed using samtools^57^; Finally, featureCounts^58^ was used to generate gene count data for downstream analyses.

#### *In vitro* experiments with the human lung cancer cell line 95-D and human normal cell lines

For *in vitro* drug treatment experiments, each cell line in the logarithmic growth phase was first adjusted to a concentration of 5×10^3^ cells/well using the corresponding medium, then inoculated into 96-well plates at a volume of 100 μL/well, and pre-cultured for 24 h to allow cell adhesion. Subsequent drug treatment used the experimental drugs BI-97C1 (CAS: 1228108-65-3), LY2606368 (CAS: 1234015-52-1), carfilzomib (CAS: 868540-17-4), and trimetrexate (CAS: 52128-35-5). All four drugs were first dissolved in dimethyl sulfoxide (DMSO) to prepare stock solutions of appropriate concentrations, then serially diluted with the corresponding medium to eight concentration gradients (0.25–32 μM). The final concentration of DMSO in all drug working solutions was ≤0.1%. Blank wells and control wells were included in the experiment, with four technical replicate wells set for each group. After drug treatment, the 96-well plates were returned to a 37°C incubator for 48 h of incubation.

After 48 h of drug incubation, 10 μL of CCK-8 reagent (Catalog No.: KR0009) was added to each well. Following thorough mixing, incubation was continued for 2 h. The absorbance (OD value) of each well was measured at a wavelength of 450 nm using a microplate reader. Cell viability was calculated using the formula: Cell viability (%) = [(OD value of drug-treated well − OD value of blank well)/(OD value of control well − OD value of blank well)] × 100%. The IC_50_ value was then determined for each drug treatment.

#### I*n vivo* antitumor experiments

Female BALB/c nude mice were randomly assigned to three groups (*n* = 8 per group): the BI-97C1 group received intraperitoneal injection of BI-97C1 at a dose of 3 mg/kg every 2 days, the trimetrexate group received intraperitoneal injection of trimetrexate at a dose of 60 mg/kg every 2 days, and the control group received intraperitoneal injection of an equivalent volume of normal saline every 2 days; all groups completed a treatment cycle consisting of 9 consecutive doses.

During the intervention period, the mouse body weight was recorded every 3 days. Tumor length (L) and width (W) were measured using a vernier caliper, with tumor volume calculated via the formula: Tumor volume = width^2^ × length × 0.5. Mice were euthanized at predefined time points, and tumor tissues as well as major organs (heart, liver, spleen, lungs, kidneys) were collected. Major organs were fixed in 4% paraformaldehyde, sectioned, and subjected to hematoxylin and eosin (H&E) staining for histological examination. For animal welfare compliance, mice were euthanized when tumor volume reached 1500 mm^3^.

#### Identification of DEGs in validated cell lines

DEGs analysis was performed using the DESeq2^59^ software package. Significant DEGs were identified with the criteria of |logFoldChange| > 0.58 and adjusted *p*-value <0.05. For the LINCS L1000 dataset,^23^ we selected drugs with a treatment duration of 48 h at a concentration of 10 μM and performed DEGs analysis using the limma package.^60^

#### Training models using the TCGA drug-patient response dataset

Patient-drug response prediction was framed as a binary classification task (responder vs. non-responder), adopting the same feature set as employed in the drug-cell sensitivity analysis. The processed TCGA patient-drug response dataset comprises 2858 patient-drug pairs, involving 94 distinct drugs and 1386 individual patients.^16^^,^^31^ Similarly, the dataset was partitioned at the patient level into a 90% training set and a 10% test set, and 5-fold cross-validation was conducted on the 90% training set for hyperparameter tuning. This partitioning strategy ensured that patient samples were mutually exclusive across all subsets.

Subsequently, Kaplan-Meier survival analysis was performed on the test set (encompassing 294 patient-drug pairs), stratifying samples based on the median of predicted response probabilities. To account for potential confounding factors, multivariate Cox proportional hazards regression was further conducted, including predicted response probability as a continuous variable along with clinical covariates (cancer type, age, sex, and tumor stage), to assess the independent prognostic value of the model predictions. All survival analyses were performed using the survfit function from the R survival package.^61^

#### Model evaluation

For different prediction objectives, we used tailored evaluation metrics: For regression tasks, the Pearson correlation coefficient (ρ) assessed predictions of gene essentiality, while the coefficient of determination (R^2^) evaluated predictions of drug-cell line sensitivity. For classification tasks that focused on binary gene essentiality labeling, we used AUC, precision, recall, and F1 score to validate performance in distinguishing “essential” and “non-essential” genes.

### Quantification and statistical analysis

In statistical analysis, Student’s *t* test was used for normally distributed data, while the Wilcoxon rank-sum test was applied for non-normally distributed data. two-way ANOVA followed by Bonferroni multiple-comparison post-test was adopted for *in vivo* animal data analysis. For data visualization, the ggplot2 package in R software was used to generate graphs.
