# Supplementary material for: Essentiality-driven prediction of anticancer drug responses in preclinical and clinical contexts
Source: iScience. 2026 Jul 27;29(8):116980. doi: 10.1016/j.isci.2026.116980 (PMC13446342; doi:10.1016/j.isci.2026.116980)
Supplement: Supplementary file 1 [file mmc1.pdf]

iScience, Volume *29*

Supplemental information

Essentiality-driven prediction of anticancer drug responses in preclinical and clinical contexts

Hongtu Cui, Xiaohui Du, Haixia Guo, Zhen Liao, Bingying Wang, Xiang Lian, Ji-Yun Zhang, Xing Lu, Dongdong Zhang, Anqiang Ye, Jing Feng, Jing Li, Zhenshun Cheng, and Feng-Biao Guo

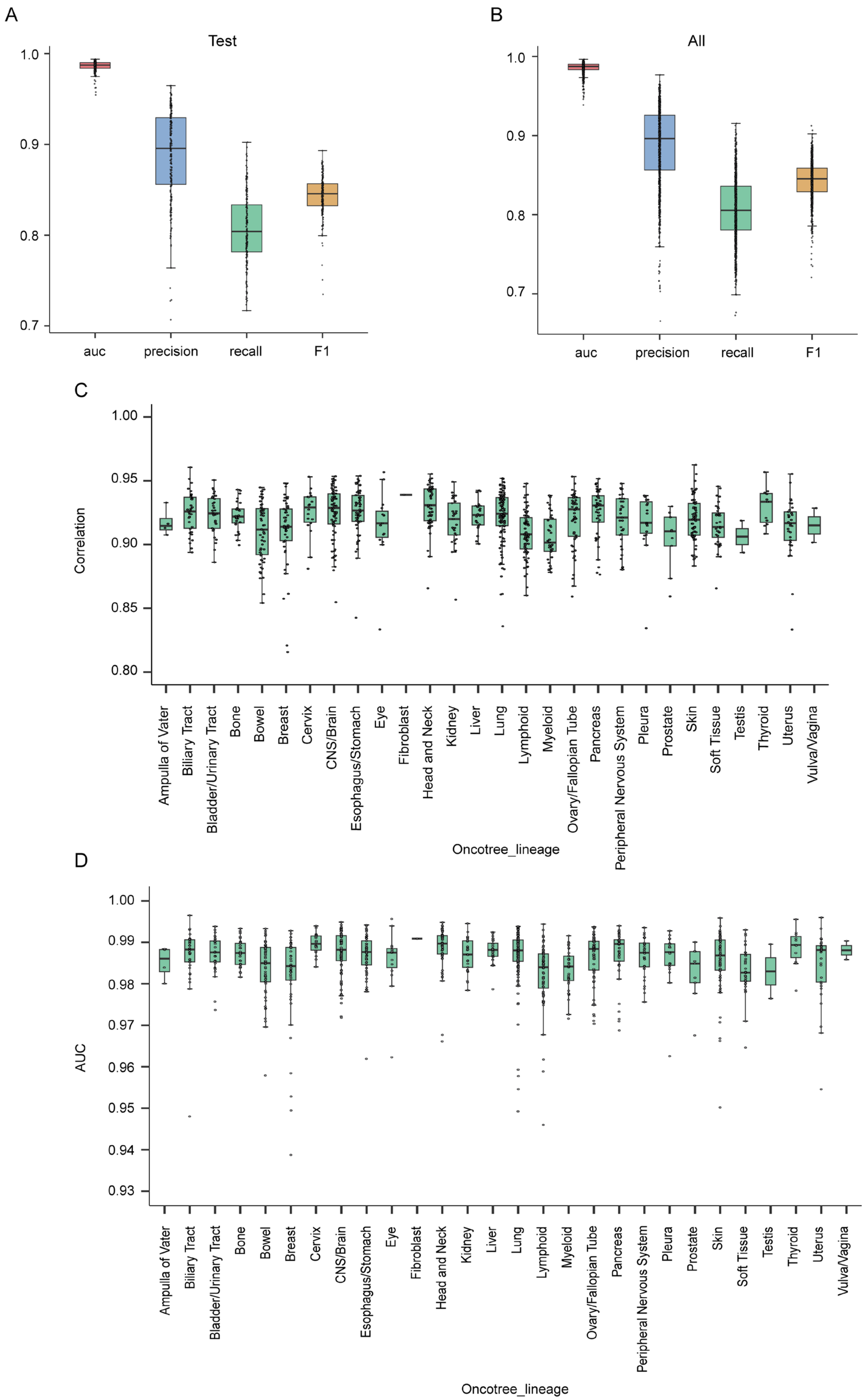
A
Test
1.0
0.9
0.8
0.7
auc
precision
recall
F1
B
All
1.0
0.9
0.8
0.7
auc
precision
recall
F1
C
Correlation
1.00
0.95
0.90
0.85
0.80
Ampulla of Vater
Biliary Tract
Bladder/Urinary Tract
Bone
Bowel
Breast
Cervix
CNS/Brain
Esophagus/Stomach
Eye
Fibroblast
Head and Neck
Kidney
Liver
Lung
Lymphoid
Myeloid
Ovary/Fallopian Tube
Pancreas
Peripheral Nervous System
Pleura
Prostate
Skin
Soft Tissue
Testis
Thyroid
Uterus
Vulva/Vagina
Oncotree_lineage
D
AUC
1.00
0.99
0.98
0.97
0.96
0.95
0.94
0.93
Ampulla of Vater
Biliary Tract
Bladder/Urinary Tract
Bone
Bowel
Breast
Cervix
CNS/Brain
Esophagus/Stomach
Eye
Fibroblast
Head and Neck
Kidney
Liver
Lung
Lymphoid
Myeloid
Ovary/Fallopian Tube
Pancreas
Peripheral Nervous System
Pleura
Prostate
Skin
Soft Tissue
Testis
Thyroid
Uterus
Vulva/Vagina
Oncotree_lineage

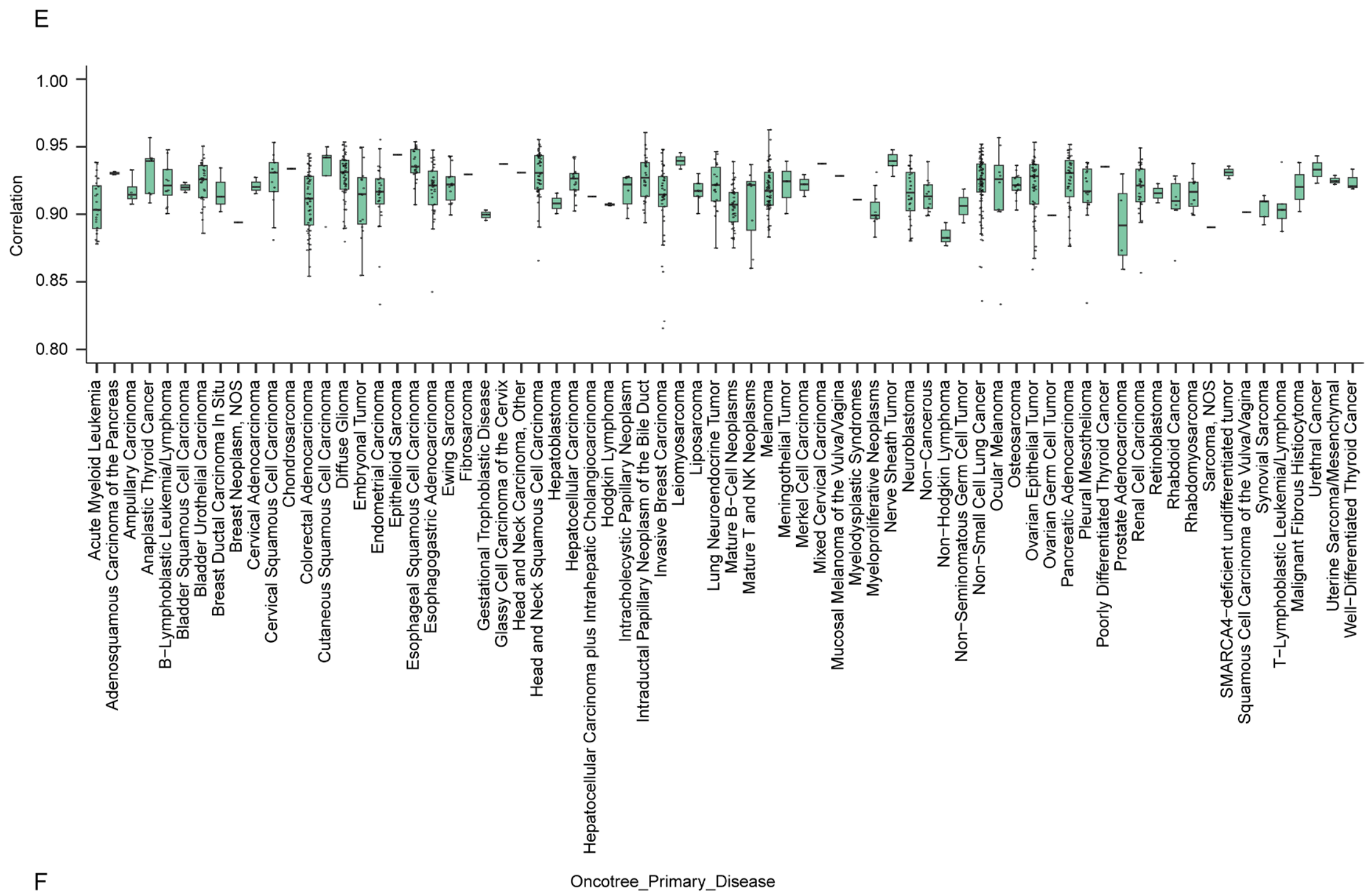

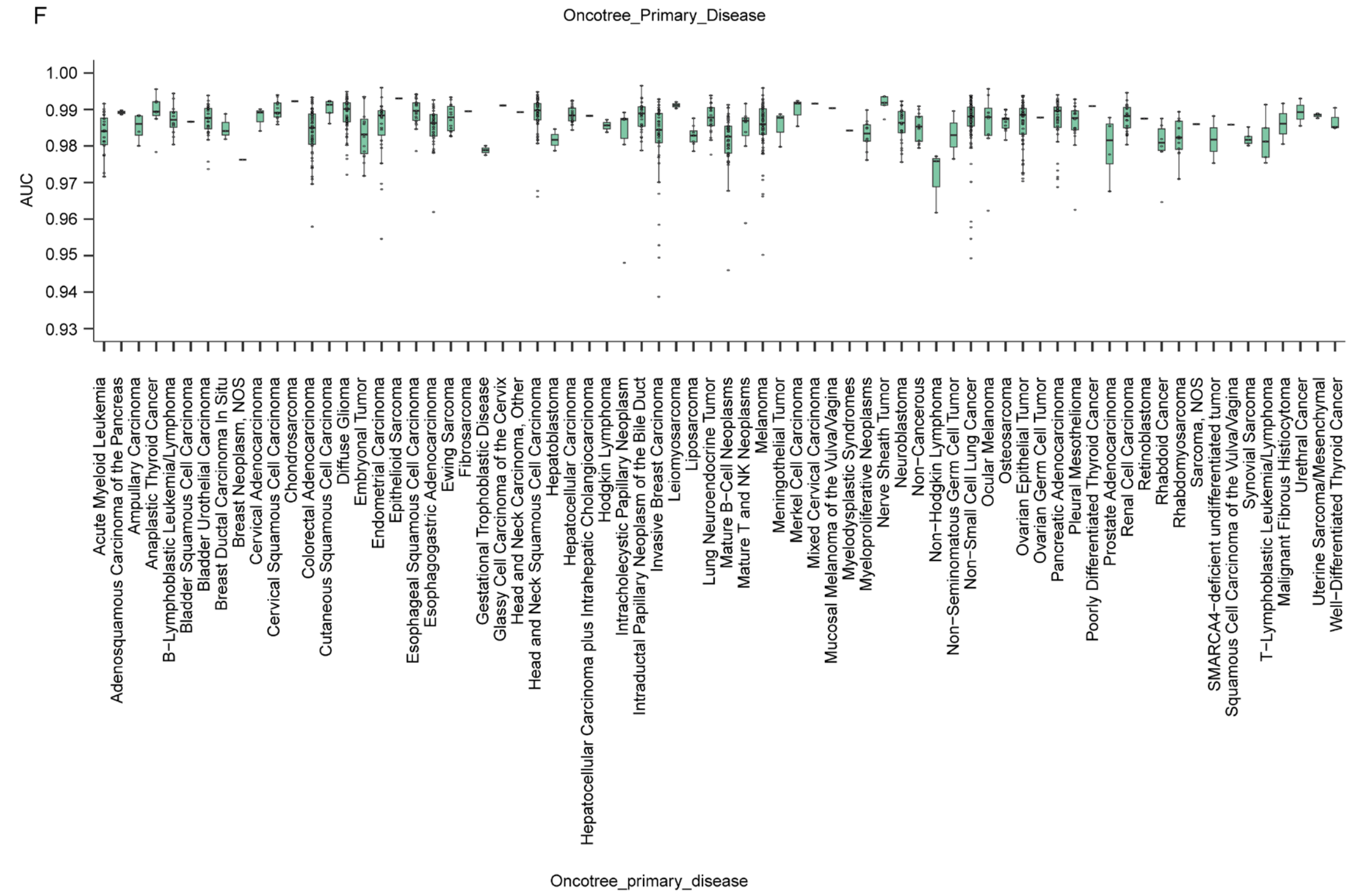

Figure S1. Performance of the WEKE Model. A. Box plot of WEKE model performance metrics (area under the curve [AUC], precision, recall, F1 score) for the test set. **B.** Box plot of WEKE performance metrics (AUC, precision, recall, F1 score) for the entire dataset. **C.** Box plot of Pearson correlation coefficients of the WEKE model for 28 tissue types (entire dataset). **D.** Box plot of AUC values of the WEKE model for 28 tissue types (entire dataset). **E.** Box plot of Pearson correlation coefficients of the WEKE model for 72 tissue types (entire dataset). **F.** Box plot of AUC values of the WEKE model for 72 tissue types (entire dataset). For all box plots, boxes indicate the interquartile range (IQR), center lines denote medians, and whiskers extend to 1.5× IQR. Data are represented as mean ± SD.

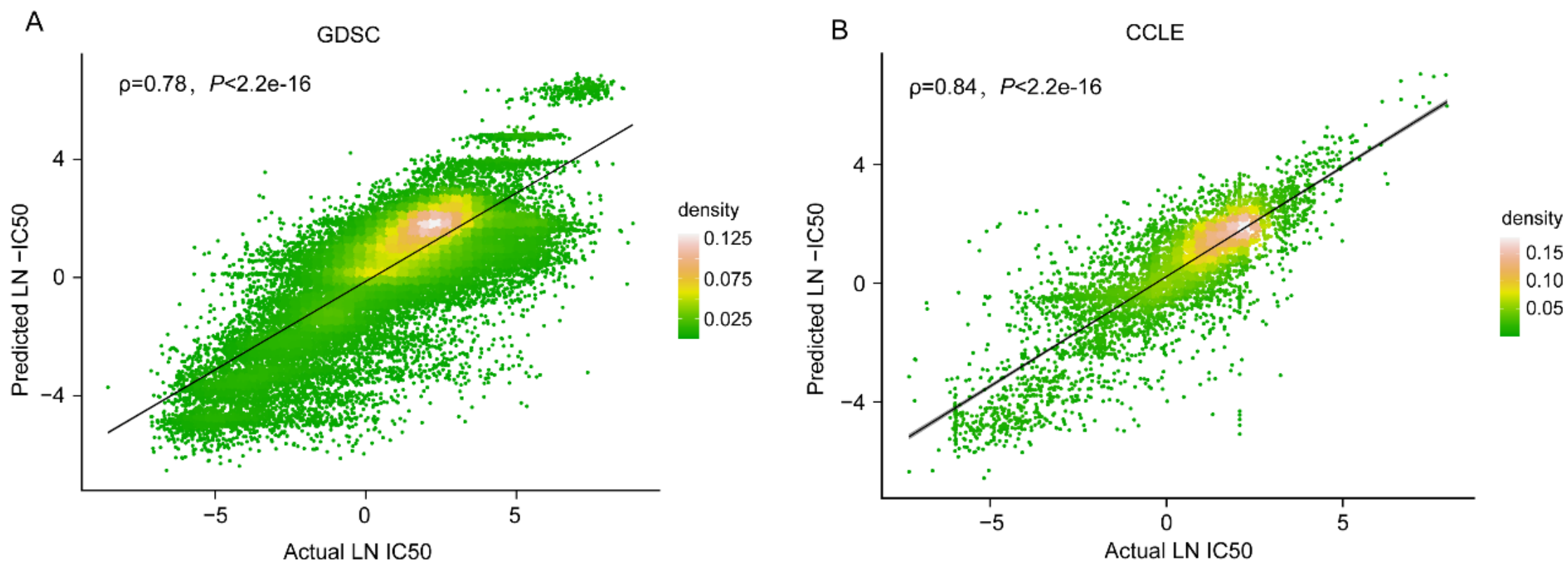

Figure S2. External validation of DeepEEAA on independent drug sensitivity datasets. The correlation between predicted and actual ln (IC50) values on the (A) GDSC and (B) CCLE datasets. Pearson correlation coefficients (ρ) and *p*-values are shown. *P*-values were calculated using a two-sided t-test.

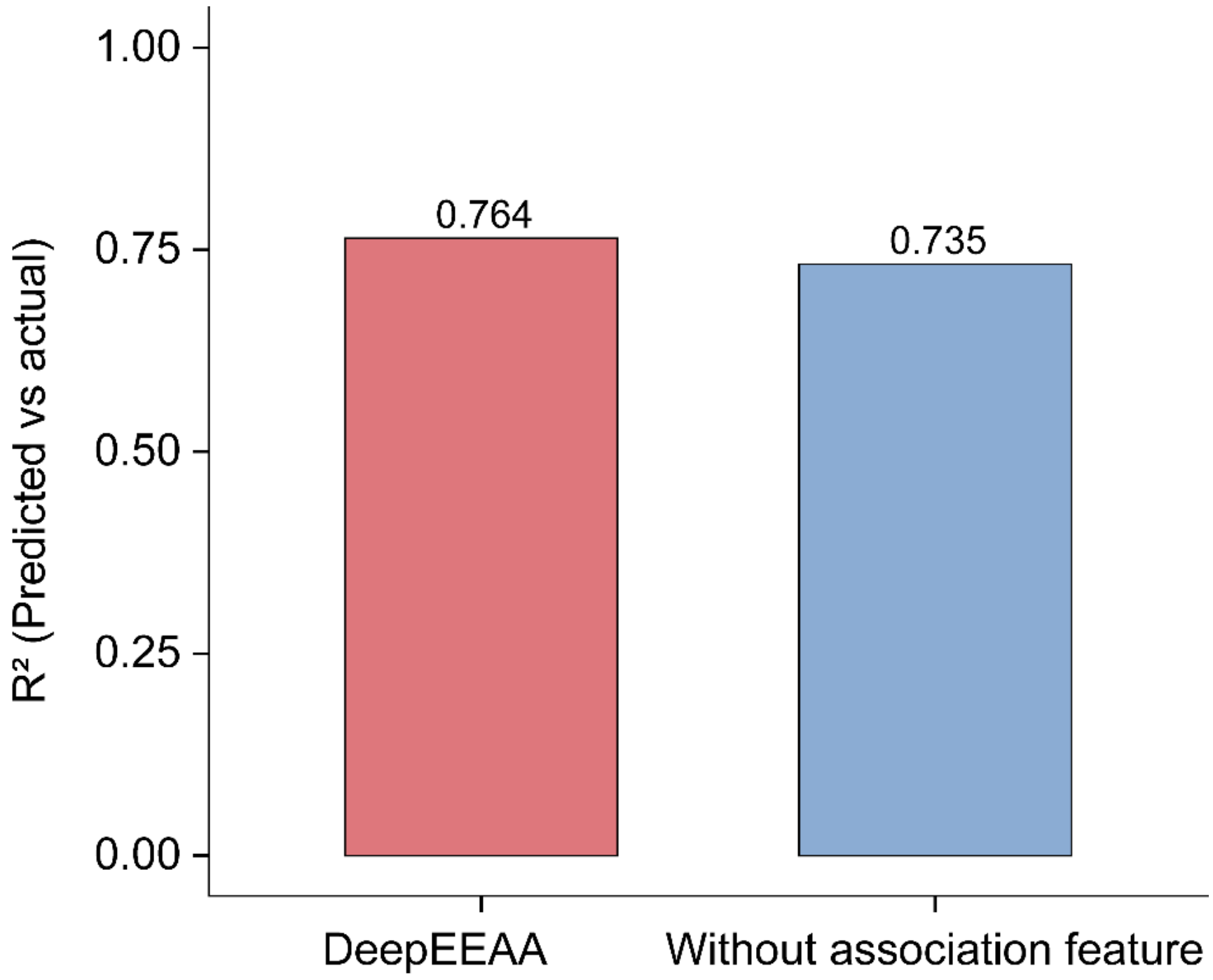

Figure S3. Ablation study of drug–gene association feature. R^2 comparison between the full DeepEEAA model and the model retrained without this feature on the same independent test set, with all other configurations unchanged. Removing them caused a drop from 0.764 to 0.735 (<3% relative decrease).

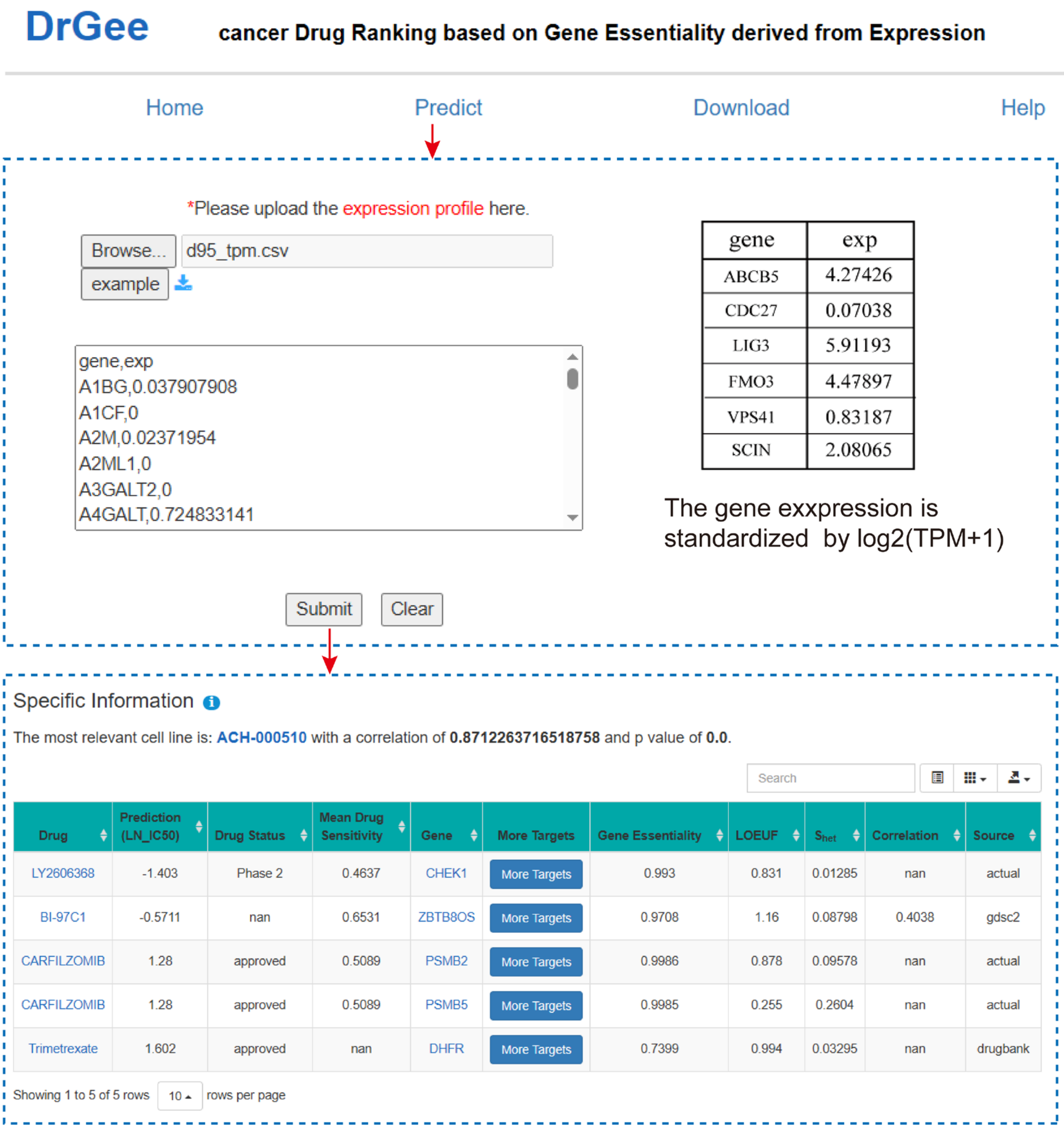

Figure S4. Schematic diagram of the DrGee online tool interface. The interface enables drug ranking based on the gene expression profile of a specific cell line. Upper panel (input): Users upload a gene expression file (e.g., tab-delimited text with gene symbols and normalized expression values). Lower panel (output): The tool returns a ranked list of candidate drugs, sorted by predicted sensitivity (lower predicted IC50 indicates higher priority), along with additional information such as inferred essential genes.

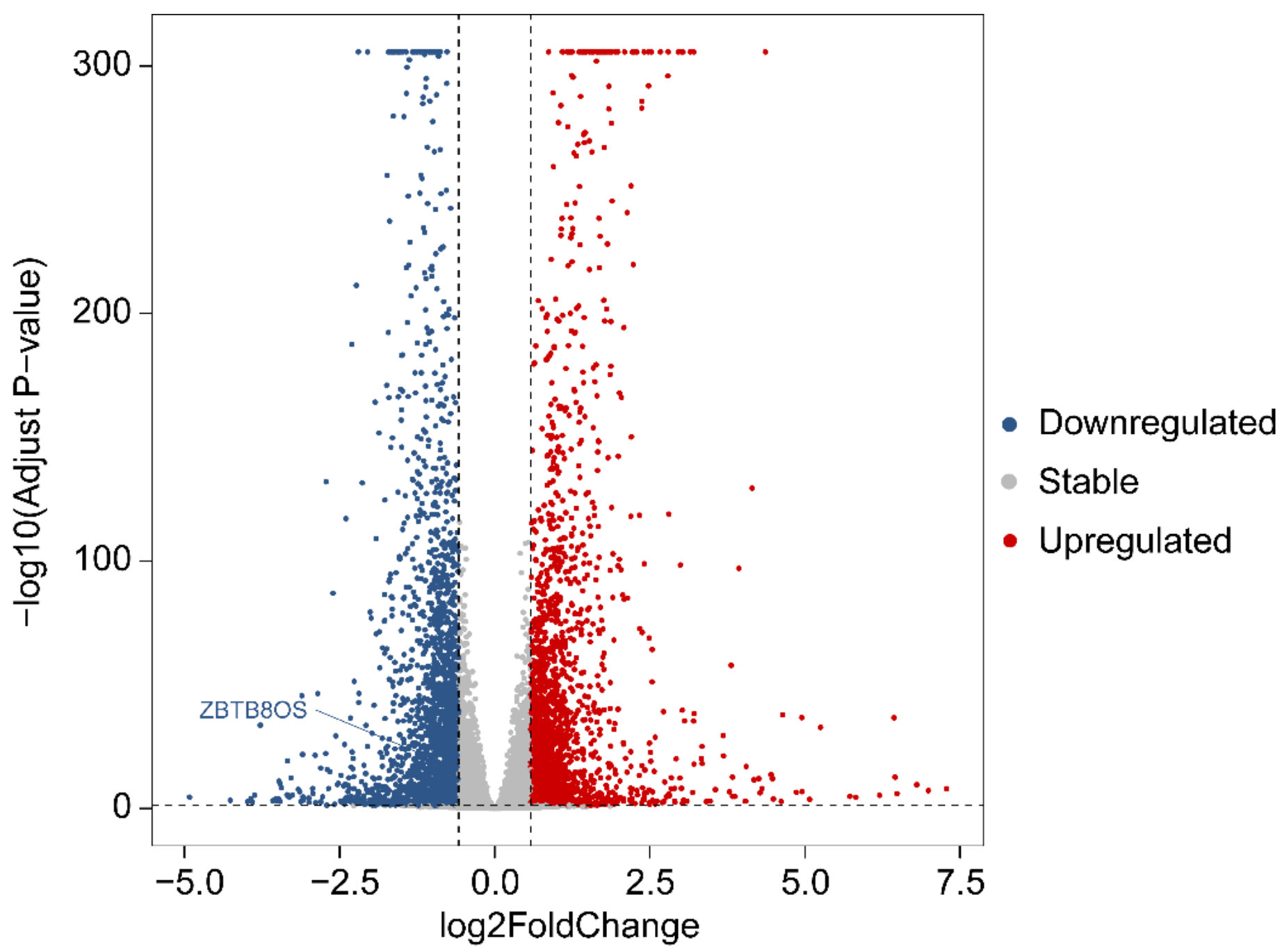

Figure S5 Volcano plot showing differentially expressed genes (DEGs) in BI-97C1-treated versus untreated 95-D cells. Genes with adjusted p-value < 0.05 and |logFoldChange| > 0.58 were considered significantly downregulated (blue) or upregulated (red); genes with no significant change were labeled as stable (gray).

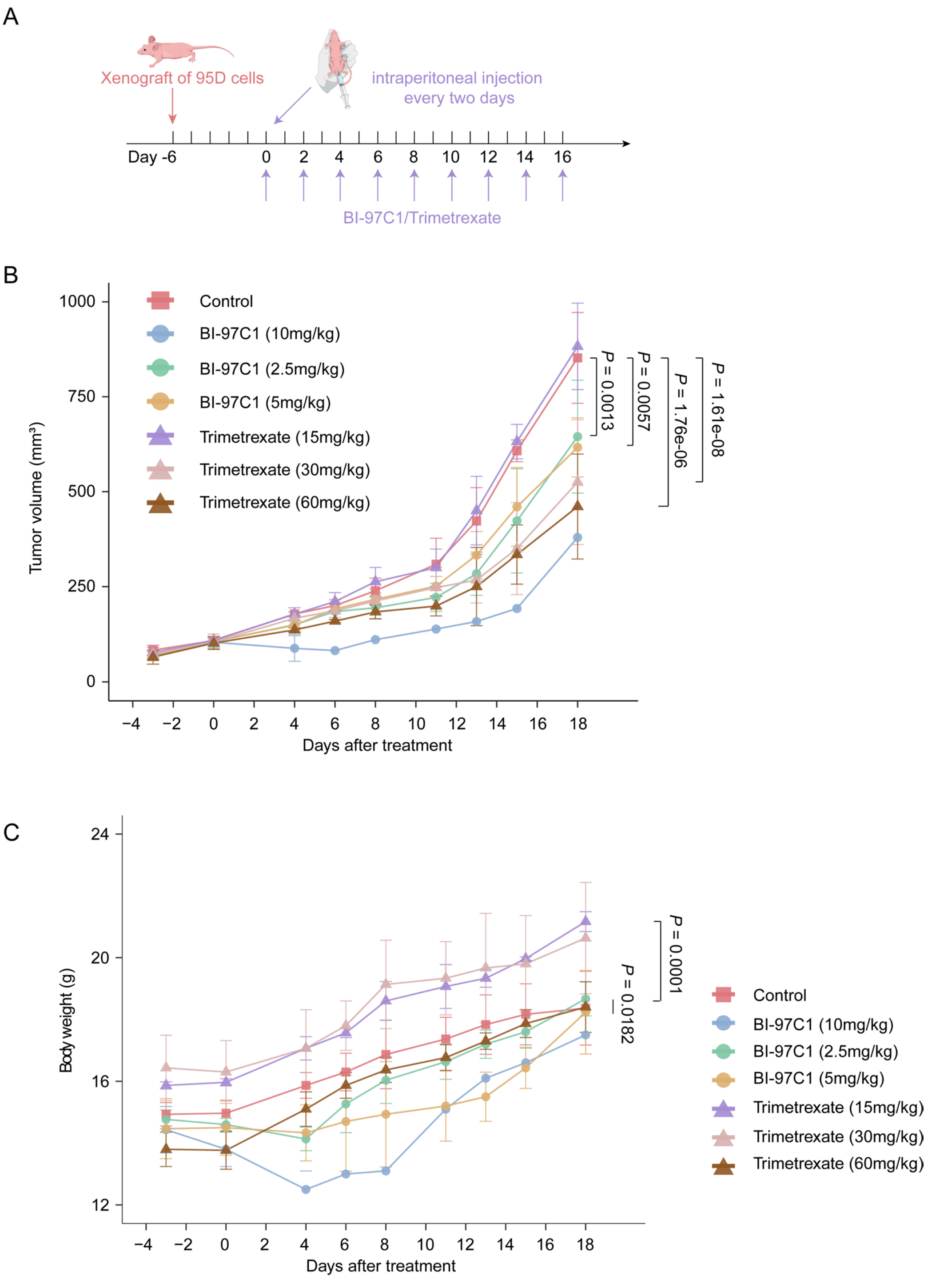

Figure S6. Preclinical Screening of Optimal Doses for BI-97C1 and Trimetrexate in Mouse 95D Cell Xenograft Tumors. A. Schematic of the antitumor experiment in 95-D tumor-bearing mice following intraperitoneal injection of saline, BI-97C1, or trimetrexate. Arrows indicate drug injection timing. **B.** Tumor volume change curves for different doses of BI-97C1 (2.5/5/10 mg/kg), trimetrexate (15/30/60 mg/kg), and the control group. **C.** Body weight change curves for mice treated with different doses of BI-97C1 (2.5/5/10 mg/kg), trimetrexate (15/30/60 mg/kg), and the control group. Data are presented as mean ± SD (n=3; two-way ANOVA followed by Bonferroni multiple-comparison post-test).

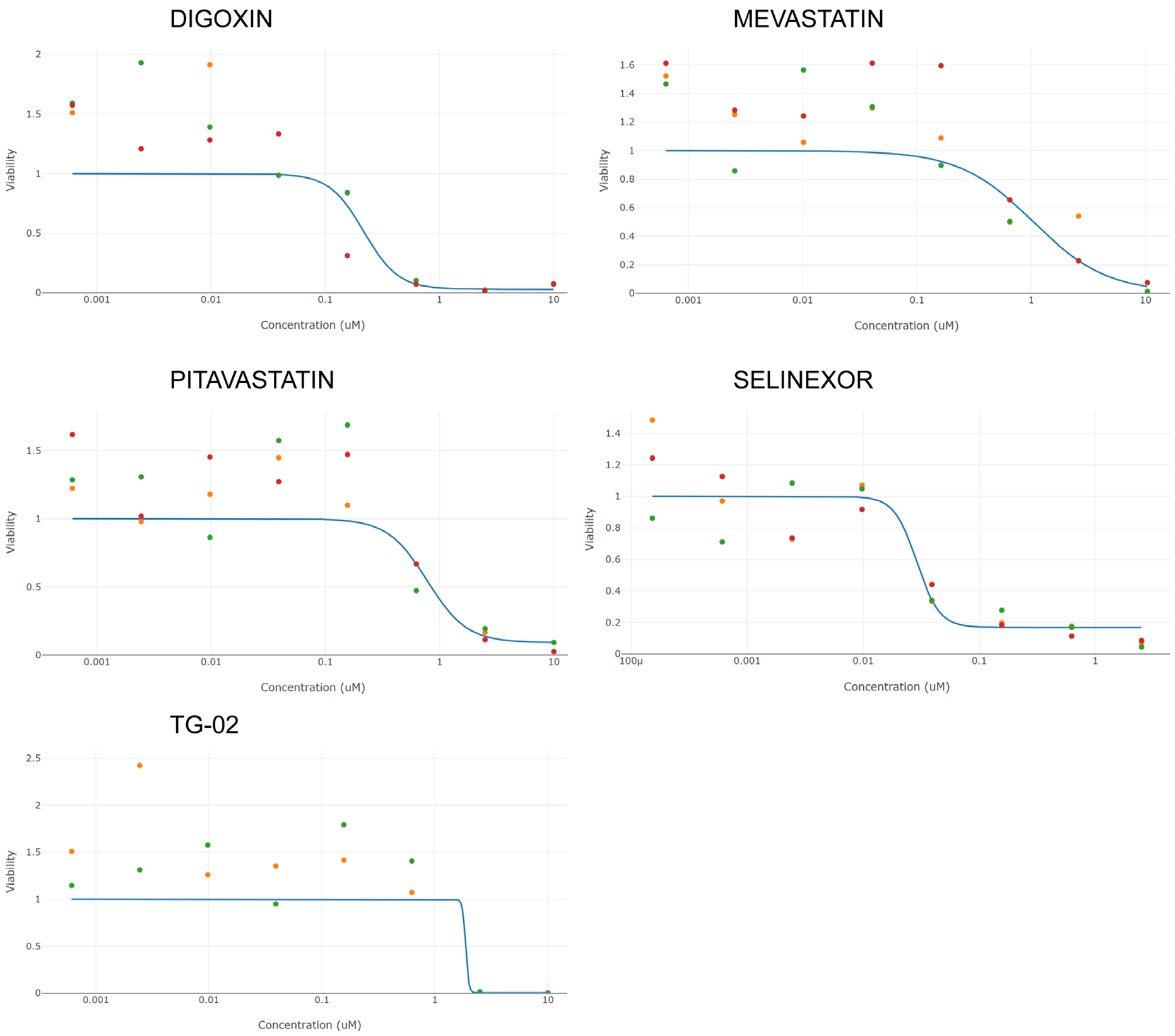

Figure S7. Dose-Response Curves of Different Drugs in the Human Ovarian Cancer OVCAR8 Cell Line from the DepMap Database. Each panel shows the dose-response relationship of the OVCAR8 cell line (from the DepMap database) to DIGOXIN, MEVASTATIN, PITAVASTATIN, SELINEXOR, and TG-02. The blue curve represents the fitted dose-response curve for each drug.

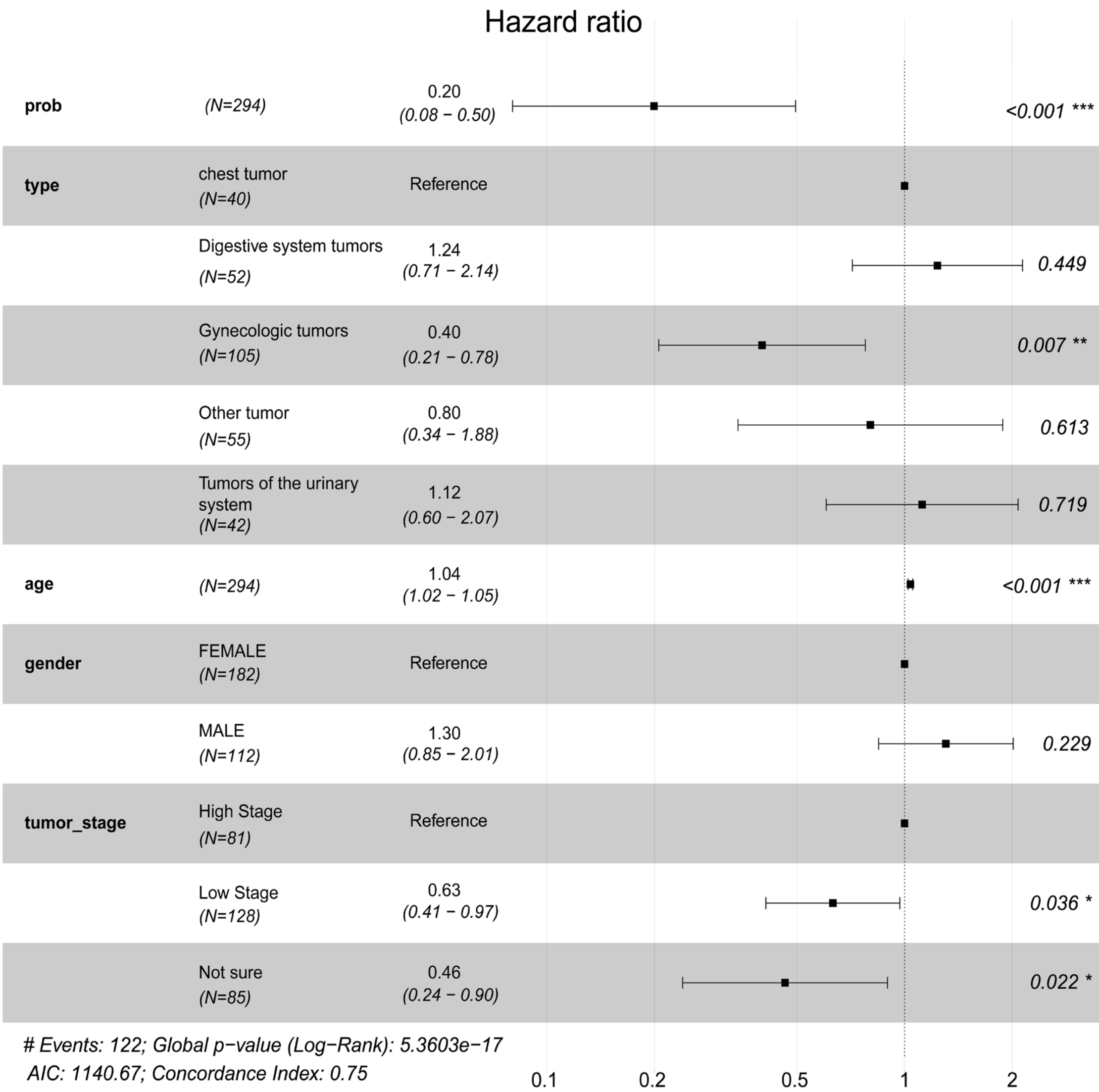

Figure S8. Forest plot of the Cox proportional hazards regression analysis for overall survival. Hazard ratios (HRs) and 95% confidence intervals (CIs) are shown for each variable, with the vertical dashed line at HR=1 representing the null effect. Chest tumor, female, and high tumor stage were set as reference groups. Statistical significance is denoted as *P<0.05, **P<0.01, ***P<0.001. The model included 294 patients with 122 events, with a global Log-rank P-value of 5.3603e-17, Akaike information criterion (AIC) of 1140.67, and concordance index (C-index) of 0.75, indicating good predictive performance.

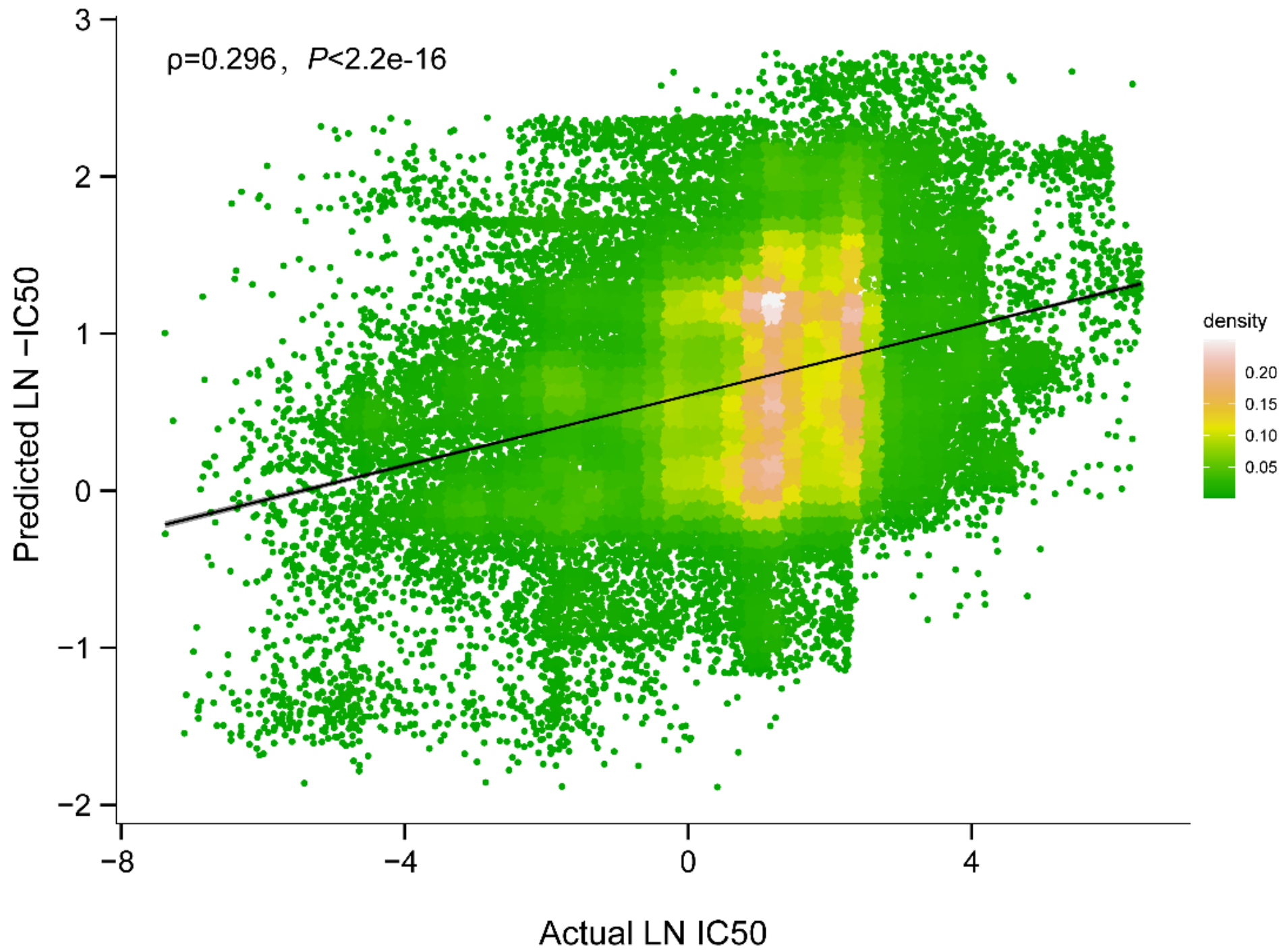

Figure S9. Scatter plot showing the drug-split performance of DeepEEAA across all cell line–drug pairs in the independent test set containing 326 drugs and 671 cell lines. *P*-values were calculated using a two-sided t-test.

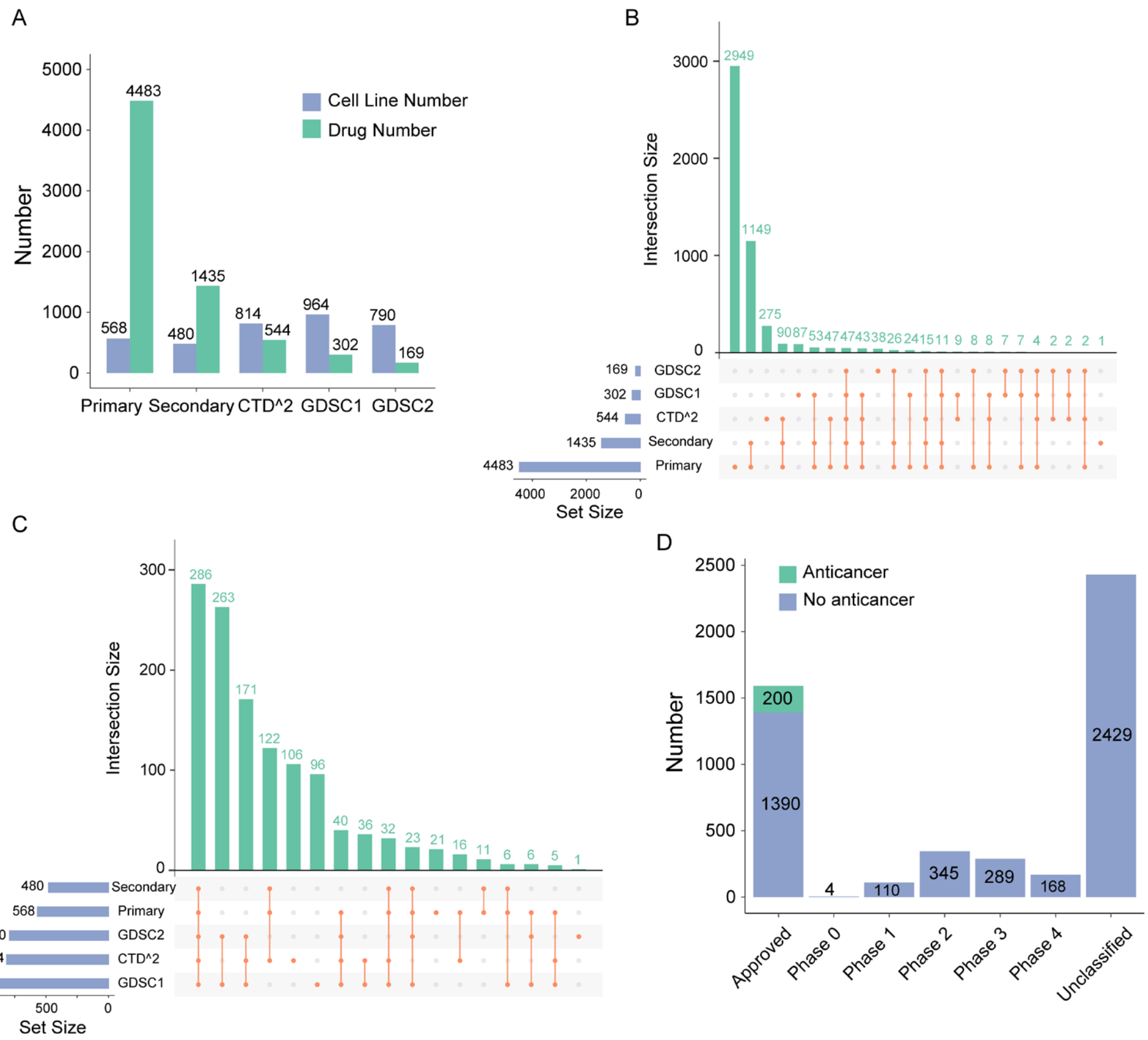

Figure S10. A. Bar chart showing the number of cell lines (blue) and drugs (green) in the Primary, Secondary, CTD², GDSC1, and GDSC2 sub-libraries of the DepMap database. **B.** Upset plots illustrating drug distribution across the aforementioned sub-libraries (GDSC2, GDSC1, CTD², Secondary, Primary), with green bars representing intersection sizes and annotations indicating the corresponding sub-libraries. **C.** Upset plots showing cell line distribution across the aforementioned sub-libraries (GDSC2, GDSC1, CTD², Secondary, Primary), with green bars indicating intersection sizes and labels for the corresponding sub-libraries. **D**. Bar chart presenting drug distribution by development stage.

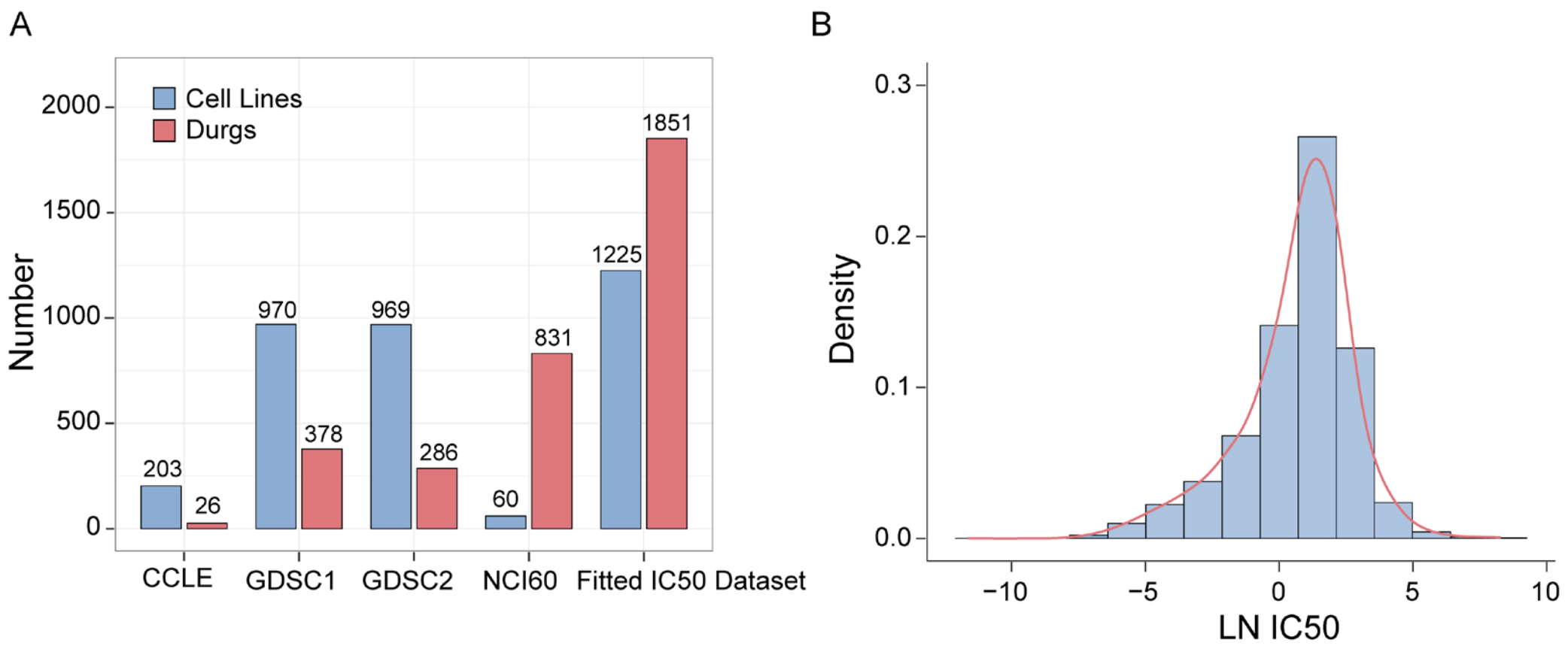

Figure S11. Distribution Characteristics of the IC_{50} Dataset Refitted Using the DepMap Database. A. Bar chart showing the number of cell lines and drugs across different sources (CCLE, GDSC1, GDSC2, NCI60) and the DepMap-refitted drug response dataset (Fitted IC_{50} Dataset). Blue bars represent cell lines; red bars represent drugs. **B.** Distribution of LN (IC_{50}) values from the DepMap-refitted drug response dataset.
